# Strategies for knowledge exchange for action to address place-based determinants of health inequalities: an umbrella review

**DOI:** 10.1093/pubmed/fdac146

**Published:** 2022-11-30

**Authors:** E Halliday, A Tompson, E McGill, M Egan, J Popay

**Affiliations:** Division of Health Research, Faculty of Health and Medicine, Lancaster University, Lancaster LA1 4YG, UK; Department of Public Health, Environments and Society, Faculty of Public Health and Policy, London School of Hygiene & Tropical Medicine, London WC1H 9SH, UK; Department of Health Services Research and Policy, London School of Hygiene & Tropical Medicine, London WC1H 9SH, UK; Department of Public Health, Environments and Society, Faculty of Public Health and Policy, London School of Hygiene & Tropical Medicine, London WC1H 9SH, UK; Division of Health Research, Faculty of Health and Medicine, Lancaster University, Lancaster LA1 4YG, UK

**Keywords:** places, social determinants, health inequalities, knowledge exchange

## Abstract

**Background:**

Place-based health inequalities persist despite decades of academics and other stakeholders generating ideas and evidence on how to reduce them. This may in part reflect a failure in effective knowledge exchange (KE). We aim to understand what KE strategies are effective in supporting actions on place-based determinants and the barriers and facilitators to this KE.

**Methods:**

An umbrella review was undertaken to identify relevant KE strategies. Systematic reviews were identified by searching academic databases (Medline, Embase, Scopus, Web of Science) and handsearching. Synthesis involved charting and thematic analysis.

**Results:**

Fourteen systematic reviews were included comprising 105 unique, relevant studies. Four approaches to KE were identified: improving access to knowledge, collaborative approaches, participatory models and KE as part of advocacy. While barriers and facilitators were reported, KE approaches were rarely evaluated for their effectiveness.

**Conclusions:**

Based on these four approaches, our review produced a framework, which may support planning of future KE strategies. The findings also suggest the importance of attending to political context, including the ways in which this may impede a more upstream place-based focus in favour of behavioural interventions and the extent that researchers are willing to engage with politicized agendas.

## Background

Place-based interventions have gained policy attention,^1^ with evidence suggesting they can be effective in improving social determinants of health.^2^ Nevertheless, place-based health inequalities persist, worsening since the COVID-19 pandemic.^3^ This has led to calls for *‘levelling up … geographical health inequalities’* (p. 16),^4^ with a recent UK Government White Paper expressing the intention to address disparities through investment in communities and places.^5^

Place-based determinants concern the physical, economic and social environments of (usually geographical) places. Strategies to improve these environments may address a range of determinants including the natural environment (e.g. green spaces); cultural and related services (e.g. leisure centres); the environment and regulation (e.g. licensing); as well as regeneration and planning, transport, the economy and community development.

Reducing place-based health inequalities through evidence-informed action has proved challenging. This may partially reflect a failure in effective knowledge exchange between researchers and other stakeholders. Firstly, there is a relatively limited body of research on interventions affecting social and economic environments compared to interventions improving the physical environment, and relatively little evidence on equity impacts.^2^ Secondly, guidance on place-based approaches emphasizes the importance of involving community knowledge and skills in planning and implementing place-based approaches to tackle health inequalities.^1^ However, the extent to which lay knowledge informs research and decision-making remains limited.^6^

Knowledge exchange (KE) encompasses the sharing of ideas, beliefs, evidence and expertise between a range of groups (e.g. publics, policy makers, practitioners).^7^^,^^8^ New understandings and practices may emerge from these interactions that could help address place-based health inequalities. We therefore aimed to understand what KE strategies are effective in supporting the design and implementation of actions on place-based determinants to address health inequalities, and the barriers and facilitators to KE.

## Methods

### Review questions

The review addressed three questions: (i) What models of KE are used in the context of place-based determinants of health inequalities? (ii) What is the effectiveness of this KE? (iii) What are the barriers and facilitators?

### Review methodology

Umbrella reviews are a method of synthesising a broad evidence-base for policy and practice.^9^ They have been used for evidence syntheses on general place-based interventions,^2^ as well as for housing,^10^ the built environment,^11^ and road safety.^12^ The methodology involves searching for, extracting, and synthesising findings from systematic review data,^9^^,^^13^ but can also involve using data from primary studies contained within these reviews.^2^^,^^12^

### Search strategy

The search strategy included a combination of terms for place-based determinants, knowledge exchange and systematic reviews. Four databases (Medline, Embase, Scopus, Web of Science) were searched in November 2020 and limited to English language publications between 1970 and 2020 (Supplementary File 1). Additionally, the bibliographies of included reviews were hand searched.

### Screening of records

The inclusion and exclusion criteria are detailed in Table 1. We used ‘knowledge exchange’ as an umbrella term recognizing this ranged from a more linear translation of (usually) research evidence to processes sharing multiple sources of knowledge (e.g. practitioners, policy makers, the public).^7^^,^^14–16^ A broad definition of knowledge beyond research evidence was considered (e.g. practitioner and lay knowledge, local contextual information). Reviews reporting advocacy and community-based participatory research were only included if KE was reported as a component.

**Table 1:** Inclusion and exclusion criteria

Inclusion criteria:	1. Develops or describes knowledge exchange model, analyses process or evaluates effectiveness of knowledge exchange or evidence use efforts in a real-world setting 2. Focus on place-based determinants of health (defined as the neighbourhoods or the physical and material environment, i ncludes: cultural services (e.g. parks, leisure centres, libraries, museums); environmental services (e.g. waste collection, food and water safety, community safety), planning and developments services (e.g. monitoring and enforcing building regulations, planning policy, economic and community development), housing, transportation and licensing) 3. Local or regional setting 4. High income countries (current OECD countries) (Australia, Austria, Belgium, Canada, Chile, Colombia, Costa Rica, Czech Republic, Denmark, Estonia, Finland, France, Germany, Greece, Hungary, Iceland, Ireland, Israel, Italy, Japan, South Korea, Latvia, Lithuania, Luxembourg, Mexico, Netherlands, New Zealand, Norway, Poland, Portugal, Slovakia, Slovenia, Spain, Sweden, Switzerland, Turkey, United Kingdom, United States) 5. Literature reviews with systematic search (in order to be included, the review authors must report searching at least 2 academic databases and clearly state inclusion/exclusion criteria). Reviews may synthesis evidence from qualitative or quantitative studies of any design 6. 1970–2020 7. English language
Exclusion criteria:	1. Review is purely theoretical/conceptual and does not report evidence of KE processes/mechanisms/effectiveness in real-world settings 2. Lifestyle determinants of health (e.g. behaviour change interventions aimed at reducing smoking) or institutional setting (e.g. schools) 3. Healthcare setting (primary, secondary or tertiary) 4. National or global setting 5. Non-systematic review 6. Grey literature, conference papers, theses and protocols

Covidence was used to manage the screening process.^17^ Two reviewers screened all titles and abstracts, with 10% of records double screened independently. Potentially relevant full paper manuscripts were considered against the inclusion criteria, with 10% of manuscripts double screened. Disagreements were resolved by consensus.

### Data extraction and quality appraisal

Data extraction sheets were developed from a previous review,^2^ and following piloting, two authors independently extracted characteristics about the included reviews and their descriptions of primary studies (Supplementary File 2). All data extraction templates were cross-checked by a second reviewer, with differences resolved through discussion with team members. Included reviews were appraised using the CASP checklist for systematic reviews,^18^ with data also extracted on the quality appraisal of individual studies, if reported by the review.

### Synthesis and reporting

Data charts related to the review questions (KE models; outcomes/effectiveness and recommendations; barriers and facilitators) were created in a spreadsheet. A thematic synthesis considered themes within and across these topics. Data related to KE was initially grouped into three broad types (i) ‘evidence access’ (KE activity to improve accessibility or the dissemination of research), (ii) ‘active KE’ (mechanisms to support the sharing and/or mobilization of knowledge) and (iii) ‘both’ (combining evidence access and active KE) before a more nuanced analysis of data was undertaken to identify different KE approaches. Our consideration of health equity included reference to the Progress-Plus factors^19^ by the included reviews.

## Results

**Fig. 1:**
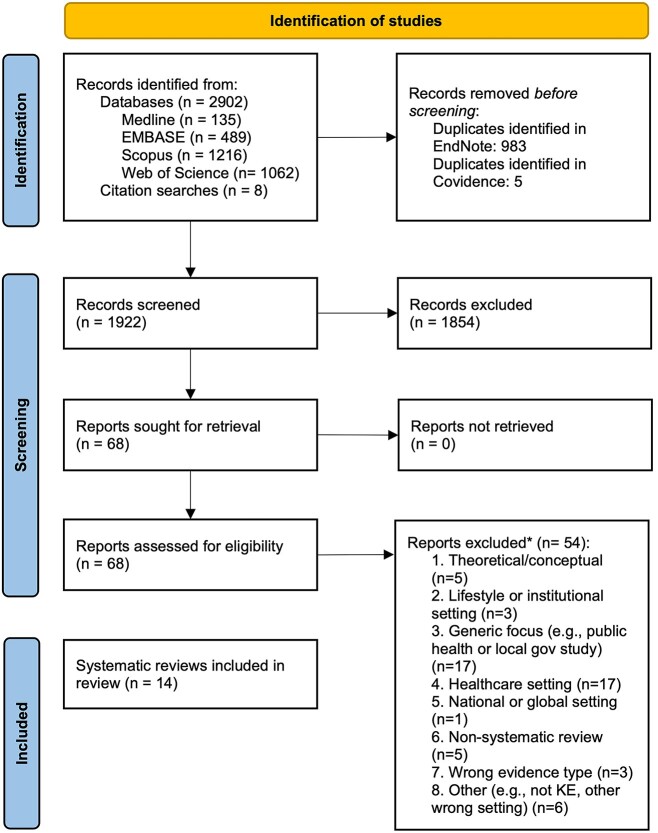
PRISMA 2020 flow diagram.

Figure 1 details the results of screening. We included 14 systematic reviews: 2 fully met the inclusion criteria and data were extracted at a review level; 12 were a partial match and the data they reported for studies matching our inclusion criteria were extracted. In total, the 14 reviews included 118 relevant primary studies and 105 unique studies.

### Summary of included systematic reviews

Characteristics of included systematic reviews and studies are summarized in Supplementary File 3a and b. The reviews were published between 2011 and 2020 and included a wide range of study designs. Six were scoping reviews and eight synthesized literature on evidence-based policy-making.

Geographical details were reported for 95 (90%) of the unique studies: Nearly 80% of these were located in three countries (USA = 44, 46%; Canada =17, 18%; UK = 14, 15%). Of the 74 studies with setting information reported, over half were based in the community (40, 54%). In terms of place-based determinants, 60 (61%) of the 99 studies with these details available addressed environmental health and contaminants, the built environment and/or planning. The remaining studies addressed a range of determinants including transport, urban regeneration, food insecurity or production, community safety, the economy, alcohol licensing, tobacco control, housing, and leisure and recreation. Fifteen studies focused on the social determinants of health. Three focused on health inequalities. The study design was reported for 53 studies; all but two used qualitative or mixed methods approaches. The results of the critical appraisal of the reviews are reported in Supplementary File 4. Five reviews reported quality appraisal of included studies.^20–24^ Two studies met all the CASP criteria.^20^^,^^23^

Key findings related to our review questions are presented below. Supplementary File 5 provides the original data extraction charts for these topics.

### Knowledge exchange approaches

**Fig. 2:**
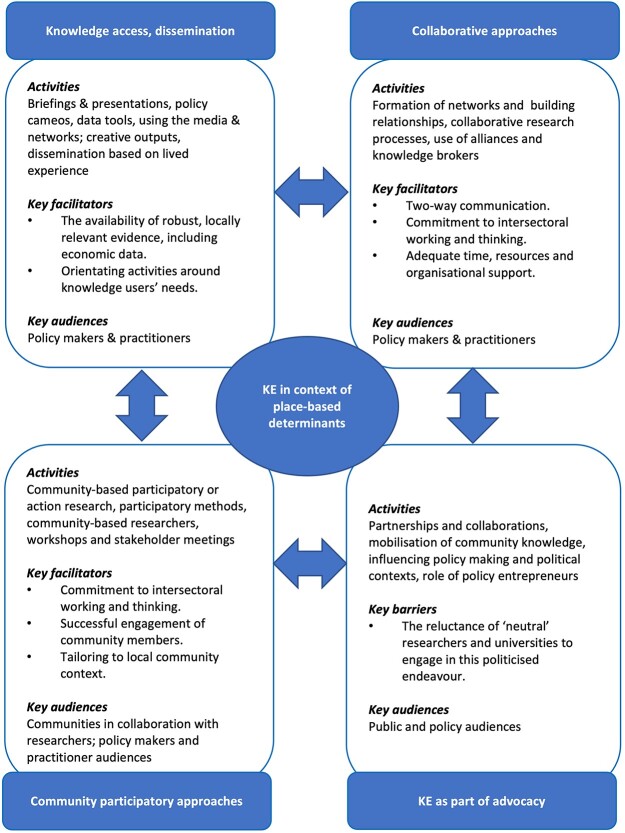
Knowledge exchange in context of place-based determinants.

Thematic analysis of data identified four KE approaches being adopted in the context of place-based determinants: (i) knowledge access and dissemination, (ii) collaborative models, (iii) community participatory strategies and (iv) KE as part of advocacy. Table 2 and Fig. 2 summarizes each of these KE approaches, detailing their activities, audiences and types of knowledge exchanged.

**Table 2:** KE approaches, audiences and knowledge types

KE approach	No of data sources	Audiences or partners for KE (*n* = reviews or primary studies)	Knowledge types (*n* = reviews or primary studies)	Study references
Knowledge access and dissemination	*n* = 22 (1 full review and 21 primary studies from 9 partial reviews)	Policy makers (*n* = 14); lay groups including Innuit people (*n* = 4); city council officials including local authority officials and Directors of Public Health (*n* = 3); local policy and programme decision makers (*n* = 2); regional government officials (*n* = 2); professionals (undefined) (*n* = 2); leaders and advocates (*n* = 1); members of tobacco control network (*n* = 1); policy advisors (*n* = 1); regional health service officials (*n* = 1); university staff (*n* = 1)	Research (*n* = 18); data (*n* = 6); policy maker and practitioner knowledge (*n* = 2); lay knowledge defined in terms of participatory research findings; Innuit people’s knowledge; lived experience of inequities (*n* = 5); other knowledge types (research or evidence undefined or ‘not academic’; needs assessments, surveys or consultations and case studies) (*n* = 8).	**Review level data:** Lorenc (2014) (2/16 studies) **Primary study references:** ARM-01; FAR-01; FAR-PLA-01; FAR-02; FAR-OLI-01; HAY-OLI-01; KNE-04; KNE-05; MAS-02; McD-02; McD-03; OLI-ORT-01; OLI-02; OLI-03; OLI-05; ORT-01; ORT-02; ORT-03; PLA-01; PLA-02; PLA-03
Collaborative models	*n* = 10 (2 full reviews and 8 primary studies from 7 partial reviews)	Policy audiences or policy makers (*n* = 6); knowledge producers, local authority and university researchers (*n* = 4); lay groups including community leaders and the Aborigine community (*n* = 4); local authority decision-makers or officers (*n* = 3); regional health service officials (*n* = 1); city council officials (*n* = 1); decision-makers (*n* = 1); knowledge consumers (not defined) (*n* = 1); advocacy groups and NGOs (*n* = 1) and other stakeholder groups (church, farmers and private sector) (*n* = 1)	Research (*n* = 8); data (*n* = 1); policy or practitioner knowledge (*n* = 2); lay knowledge defined in terms of community or traditional knowledge (*n* = 3); other knowledge types (evaluative evidence beyond academic studies and other information or evidence not defined) (*n* = 5)	**Review level data:** Lorenc (2014) (2/16 studies); Wine (2017) (6/45 studies excluding duplicates) **Primary study references:** COH-05; COH-06; HAY-OLI-01; KNE-01; KNE-03; MAS-02; OLI-WIN-01; ORT-01
Community participatory approaches	*n* = 16 (1 full review and 15 primary studies from 7 partial reviews)	Lay groups in collaboration with researchers, policy makers and practitioners (*n* = 9); lay groups including Innuit people (*n* = 2) and Aborigine community (*n* = 1); public and policy audiences (*n* = 1); neighbourhood residents and researchers (*n* = 1); policy makers (*n* = 1); knowledge producers and consumers (*n* = 2)	Research evidence (*n* = 14); data (*n* = 10); policy or practitioner knowledge (*n* = 7); lay knowledge defined in terms of community knowledge, participatory research findings, Innuit people’s knowledge and lived experience (*n* = 16); other knowledge types (impact assessments and formalized knowledge) (*n* = 11)	**Review level data:** Wine (2017) (22/45 studies excluding duplicates) **Primary study references:** COH-04; FAR-02; McD-02; McD-03; OLI-WIN-01; PLA-02; PLA-03; PLA-05; SAL-01; SAL-WIN-01; SAL-02; SAL-WIN-02; SAL-WIN-03; SAL-03; SAL-04
KE as part of advocacy	*n* = 13 (13 primary studies from 3 partial reviews)	Public and policy audiences (*n* = 7); policy makers (*n* = 3); policy makers, professionals and public (*n* = 3)	Research evidence (*n* = 7); data (*n* = 6); policy or practitioner knowledge (*n* = 1); lay knowledge defined in terms of community knowledge and lived experience (*n* = 8); other knowledge types (impact or needs assessments, reports, commission findings and research in general) (*n* = 8)	**Review level data:** n/a **Primary study references:** COH-PLA-01; COH-01; COH-02; COH-03; COH-04; COH-05; COH-06 FAR-PLA-01; FAR-03; FAR-04; PLA-03; PLA-04; PLA-05

One full review^21^ and 21 primary studies^25–45^ reported on knowledge access and dissemination with primarily policy and practice audiences. Two full reviews^21^^,^^46^ and eight primary studies^30^^,^^33^^,^^40^^,^^47–51^ reported collaborative KE models. This approach aimed to facilitate engagement and interaction during research processes involving researchers and other knowledge users, primarily public sector stakeholders. One full review^46^ and 15 primary studies^27^^,^^34^^,^^35^^,^^44^^,^^45^^,^^51–60^ reported on community participatory approaches, including participatory or action research^34^^,^^46^^,^^52^^,^^55–60^ to enable lived experiences of inequalities to influence decision making and action. Thirteen studies^29^^,^^45^^,^^47^^,^^48^^,^^52^^,^^53^^,^^61–67^ reported using KE approaches as a mechanism for advocacy to raise the local profile of social determinants of health and health inequalities among public, professional and policy stakeholders.

### Effectiveness of KE

There was little empirical evidence reported on the impact of any KE approach. A handful of studies of collaborative models and community participatory approaches provided positive appraisals of KE based on qualitative insights. For example, a case study of the ‘Healthy in the City’ programme described how collaboration paved the way for further research-policy partnerships.^20^ Further impacts of collaborative approaches included fostering stakeholder engagement, improved communication and better meeting the evidence needs of public health decision-makers,^49^ also enabling capitalization of funding opportunities for future research.^50^ In the context of community participatory approaches, the development of trusting relationships improved the validity and relevance of research, thus increasing its potential for local uptake.^34^ Cohen provided a rare report of a public health advocacy campaign impacting place-based determinants of health.^61^^,^^68^ As a consequence, the Fresh Food Financing Initiative was formed and 32 supermarkets were built in low-income neighbourhoods. However, it was unclear how this campaign was evaluated. Studies of knowledge access and dissemination were not evaluated, but hypothesized that using evidence, especially robust scientific research, would result in an improvement in policy-making.

### Equity consideration in studies

Eight studies of knowledge access and dissemination included an explicit health equity focus.^26–29^^,^^41^^,^^43–45^ All but one study^41^ were from two reviews about knowledge to action strategies to tackle health inequalities.^24^^,^^68^ The majority (*n* = 11) of studies reporting community participatory approaches and all studies (*n* = 13) reporting advocacy had an explicit focus on using KE to address health inequalities. Community-based participatory research aimed to enable knowledge of local people with first-hand experience to influence research and action,^27^ and was a mechanism for utilising lay experience in efforts to tackle neighbourhood stressors.^56^^,^^57^^,^^60^^,^^69^ Involving stakeholders with experience of the setting that was the focus of the influence (e.g. political contexts) was also cited in context of advocacy work.^29^^,^^63^ Through stakeholders’ knowledge of organizational structures and hierarches, the political will to tackle health equity might be fostered. No reviews reported on Progress-plus factors although the tool was recommended in one study as a framework for considering differential outcomes.^28^

### Barriers and facilitators

Reviews were more likely to report on the barriers and facilitators of KE rather than effectiveness. Information extracted on barriers and facilitators was organized around characteristics of: (i) the individuals involved, (ii) the knowledge being exchanged, (iii) the relationships between individuals and (iv) their organizations (Supplementary File 6).

Decision makers reported the importance of the availability of high-quality, timely information that was relevant to the local context^31^^,^^33^^,^^50^^,^^70–72^ with economic evaluation of particular value.^28^^,^^32^^,^^49^^,^^71^ Activities that facilitated access to relevant research findings, included presenting evidence in forms and formats that met users’ needs—including implementable actions—and fostering long lasting relationships between researchers and policymakers.

In addition to the characteristics of the knowledge being exchanged, studies reporting collaborative models identified the need to foster two-way communication and intersectoral working and thinking in the teams involved,^30^^,^^50^ acknowledging that such approaches may take longer and require adequate resources.^49^^,^^50^ In collaborative approaches, the neutrality of the researcher added to their credibility and the usefulness of the evidence they presented.^21^ However, this neutrality could impede researchers’ willingness to engage in more politicized endeavours such as using research for advocacy.^63^^,^^73^

Limited data were available for barriers and facilitators associated with community participatory approaches, although this was reliant on the engagement and recruitment of community members.^27^^,^^34^ Studies included in a review on Inuit communities reported tailoring the entire KE process to the community context as a facilitator, in part to ensure the relevance and uptake of the resulting research.^34^ Community health workers were proposed as playing a role in supporting such activities.^52^

A common theme across KE approaches was the political context, either at the organizational or a broader, macro level. This spanned the political affiliations and ideologies of the actors involved, organizational structures and policies regarding the use of different forms of knowledge, and the prevailing political environment and its (in)stability.^22^^,^^73^ Efforts to make the case for actions on social determinants of health (and redirect resources towards this) sometimes met resistance from stakeholders favouring behavioural or lifestyle interventions.^29^ Political factors also influenced the use of evidence in organizational contexts.^31^^,^^32^ In one study, evidence was deemed ‘irrelevant’ where aspirations for local action already aligned with political ideology.^32^ Although in another study, where evidence was aligned to political ideology, the evidence was considered salient by decision makers.^31^

## Discussion

### Main finding of this study

Review-level evidence on KE and place-based determinants was relatively limited as most reviews available were only of partial relevance to our topic. Studies addressed a range of determinants, but reflecting evidence on place-based interventions, many involved KE in the context of environmental determinants. Nevertheless, our synthesis has located four KE approaches used to address place-based determinants: improving access to and dissemination of knowledge; collaborative processes typically with practitioner and policy audiences; participatory approaches with communities and the use of KE in advocacy efforts on social determinants of health and health inequalities. Consideration of health inequalities and the inclusion of lay knowledge was most evident in approaches aligned with social justice or political agendas (advocacy and community participatory approaches). While a range of barriers and facilitators were identified, a reoccurring factor across approaches was the political context within which KE occurs. There was a paucity of evidence on the effectiveness of KE.

### What is already known on this topic

Reviews of KE strategies have been published in recent years^74^^,^^75^ but reflecting the findings of this review, they have reported little evaluative evidence of the extent that KE leads to changes in knowledge or practice or affects health outcomes,^74^ with the effectiveness of KE models or frameworks often not tested or evaluated in real life contexts.^75^ Rationales cited for this gap relate to a lack of robust study designs, with the absence of shared evaluation frameworks also making it difficult to robustly compare effectiveness across studies.^20^ KE reviews in the context of advocacy also drew attention to the complexity of evaluating its underlying change mechanisms, as well as a general paucity of advocacy evaluations.^68^^,^^73^

### What this study adds

Our review identified a range of KE approaches used in place-based contexts. We found different purposes associated with these approaches ranging from facilitating access to knowledge and collaborative approaches that improve the relevance and uptake of research in decision-making, to ‘bottom up’ approaches concerned with mobilizing lived experiences of inequalities and/or advocating for change. Figure 2 provides a framework based on review findings, which could be used to support research teams in planning their approach to KE for place-based public health. Although evidence on the effectiveness of these KE types was limited, it was evident that different KE types may be used with different audiences for different purposes. ‘Knowledge access and dissemination’ and ‘collaborative models’ were direct KE mechanisms aimed at policy makers and practitioner audiences often concerned with access to relevant and timely evidence to inform decisions. ‘Participatory approaches’ typically involved collaboration between communities and researchers, serving as a mechanism for lay knowledge to influence policy and practice decisions. This was particularly the case when there was a perceived equity issue and communities collaborating with researchers sought to take action to mitigate against these (e.g. poor air quality). Similarly, the use of KE in advocacy was often politically motivated, aimed at raising awareness among both public and policy audiences.

While some barriers and facilitators were salient to particular KE approaches, the synthesis suggested that the political context was evident regardless of KE approach. In place-based settings, this may manifest, for example, in the extent that an issue is politically emotive (e.g. vehicle parking charges),^76^ or where KE concerns action on structural causes of inequalities within decision-making contexts that favour behavioural or individualistic models.^77^^,^^78^ KE approaches linked to advocacy could also present a tension. As Farrier argues, advocacy is ‘an ethical concept, grounded in the principle of distributive justice and connected to a field of research that is “unavoidably politicized’.” (p. 394)^73^ Researchers’ willingness to engage in such activities may, in part, depend on whether they view their role as an impartial one, principally concerned with evidence-informed decision making, or align themselves with political and social engagement to challenge inequalities.^79^

Finally, equity sensitive KE was most explicit in approaches underpinned by community participation and advocacy but less visible in KE approaches in organizational or professional settings. When coupled with the finding from McGowan *et al*.’s review, which identified little focus on health inequalities in place-based intervention studies,^2^ there is an urgent need for health equity to be more systematically considered by place-based public health researchers. An equity lens can support the production and application of knowledge more likely to contribute to tackling structural inequalities.^80^ It can also mitigate against KE stereotyping groups through attention to language,^81^ and by attending to power sharing between different stakeholders.^46^

### Limitations of this study

Only reviews in the peer-reviewed academic literature were included. A considerable body of grey literature on KE exists. Including this may have provided further insights into KE including its practices, audiences and impacts. We also found that definitions of ‘local’ varied considerably between contexts. We included studies with a sub-national setting, but this could cover vastly different geographies depending on country. Due to resource constraints, we extracted data from the included reviews rather than consulting the primary studies they cited. We were reliant, therefore, on the availability of non-aggregated information in the reviews, which varied considerably. One resulting gap was the ability to fully compare different types of KE, which could be the focus of future research. As done elsewhere,^22^^,^^82^ our synthesis used in part a barriers and facilitators approach to understanding KE. This has been critiqued for oversimplifying complex interactions, identities and processes^20^ and causing process models to be overlooked.^83^

## Conclusion

Our review identified a range of KE approaches and developed a framework, which could support planning of KE in the context of place-based public health. Further research is needed to understand the effectiveness of these approaches. There is a larger body of evidence on barriers and facilitators to KE, with the political context identified as a common factor. This influenced how evidence was used in decision-making and the extent to which place-based public health translates into a preference for behavioural solutions over upstream approaches.

## Supplementary Material

Supplementary_file_1_Search_Strategy_fdac146

Supplementary_file_2_-_Data_extraction_form_fdac146

Supplementary_file_3a_-_Review_Characteristics_fdac146

Supplementary_file_4_-_CASP_findings_fdac146

Supplementary_File_5_-_Data_extraction_charts_fdac146

Supplementary_file_6_-_Barriers_and_Facilitators_to_KE_fdac146

Supplementary_file_3b_-_Included_Study_Characteristics_fdac146

## Data Availability

All data generated or analyzed during this study are included in this published article (and its supplementary files).

## Notes

**E. Halliday**, Senior Research Fellow

**A. Tompson**, Research Fellow

**E. McGill**, Assistant Professor

**M. Egan**, Professor

**J. Popay**, Distinguished Professor
