# Supplementary material for: Strategies for knowledge exchange for action to address place-based determinants of health inequalities: an umbrella review
Source: J Public Health (Oxf). 2022 Nov 30;45(3):e467–77. doi: 10.1093/pubmed/fdac146 (PMC10470361; doi:10.1093/pubmed/fdac146)
Supplement: Supplementary_file_1_Search_Strategy_fdac146 [file supplementary_file_1_search_strategy_fdac146.docx]

**Supplementary file 1: Search strategy**

Databases: Medline, Embase, Scopus, Web of Science

Key terms for database searching:

1) Public health OR population health OR community health OR health inequality OR health inequity OR place-based OR community empowerment OR community asset* OR asset-based approach* OR upstream determinants OR social determinants OR health promotion OR "well being" OR wellbeing OR welfare OR benefits OR "social determinant" OR crime OR “community safety” OR transport OR planning W/3 town OR planning W/3 city OR planning W/3 neighborhood OR planning W/3 urban OR renewal W/3 town OR renewal W/3 city OR renewal W/3 neighborhood OR renewal W/3 urban OR redevelopment W/3 town OR redevelopment W/3 city OR redevelopment W/3 neighborhood OR redevelopment W/3 urban OR regeneration W/3 town OR regeneration W/3 city OR regeneration W/3 neighborhood OR regeneration W/3 urban OR revitali#ation W/3 town OR revitali#ation W/3 city OR revitali#ation W/3 neighborhood OR revitali#ation W/3 urban OR “urban health” OR housing W/3 improvement OR home W/3 improvement OR rehousing OR “housing refurbishment” OR “home refurbishment” OR “housing modification” OR “home modification” OR “healthy home” OR 4 “healthy housing” OR “affordable housing” OR “affordable home” OR “housing intervention” OR education OR “whole school” OR school W/3 environment OR greenspace or local government OR local authority OR council

2) Knowledge exchange OR knowledge transfer OR knowledge translation OR knowledge dissemination OR knowledge diffusion OR knowledge use OR knowledge utilis/zation OR knowledge management OR knowledge adoption OR knowledge uptake OR evidence use OR evidence utilis/zation

3) Systematic review OR literature review OR evidence review OR narrative review OR scoping review OR critical review OR rapid review OR umbrella review OR meta-analysis OR meta-ethnography

Searches run 24/11/2020

Medline: 1 AND 2 AND 3= 135

1) Public health OR population health OR community health OR health inequalit* OR health inequit* OR well-being OR wellbeing OR place-based OR community empowerment OR community transformation OR community asset* OR asset-based approach* OR upstream determinant* OR social determinant* OR health promotion OR welfare OR benefits OR crime OR community safety OR transport OR housing OR planning adj3 town OR planning adj3 city OR planning adj3 neighbo?rhood OR planning adj3 urban OR renewal adj3 town OR renewal adj3city OR renewal adj3 neighbo?rhood OR renewal adj3 urban OR development adj3 town OR development adj3 city OR development adj3 neighbo?rhood OR development adj3 urban OR regeneration adj3 town OR regeneration adj3 city OR regeneration adj3 neighbo?rhood OR regeneration adj3 urban OR revitali#ation adj3 town OR revitali#ation adj3 city OR revitali#ation adj3 neighbo?rhood OR revitali#ation adj3 urban OR urban health OR housing adj3 improvement OR home adj3 improvement OR affordable housing OR affordable home* OR housing intervention OR education OR whole school OR school adj3 environment OR greenspace or local government OR local authorit* OR council OR Public health/ OR Population Health/ OR Mental health/ OR Health promotion/ OR Social Determinants of Health/ OR Housing/ OR Crime/ OR Violence/ OR city planning/ OR urban renewal/ OR urban health/ OR Schools/ OR Education/ OR Local government/ /Limit to English language 314,442

2) Knowledge exchange OR knowledge transfer OR knowledge translation OR knowledge dissemination OR knowledge diffusion OR knowledge utili#ation OR knowledge management OR knowledge adoption OR knowledge uptake OR evidence utili#ation OR “evidence use” OR “knowledge use” OR “research evidence” OR Knowledge management/ Limit to English language 10,269

3) Systematic review OR literature review OR evidence review OR narrative review OR scoping review OR critical review OR rapid review OR umbrella review OR “review of reviews” OR meta-analysis OR meta-ethnography OR Systematic review/ OR Meta-Analysis/ /Limit to English language 312,259

EMBASE: 1 AND 2 AND 3 = 489

1) Public health OR population health OR community health OR health inequalit* OR health inequit* OR well-being OR wellbeing OR place-based OR community empowerment OR community transformation OR community asset* OR asset-based approach* OR upstream determinant* OR social determinant* OR health promotion OR welfare OR benefits OR crime OR community safety OR transport OR housing OR planning adj3 town OR planning adj3 city OR planning adj3 neighbo?rhood OR planning adj3 urban OR renewal adj3 town OR renewal adj3city OR renewal adj3 neighbo?rhood OR renewal adj3 urban OR development adj3 town OR development adj3 city OR development adj3 neighbo?rhood OR development adj3 urban OR regeneration adj3 town OR regeneration adj3 city OR regeneration adj3 neighbo?rhood OR regeneration adj3 urban OR revitali#ation adj3 town OR revitali#ation adj3 city OR revitali#ation adj3 neighbo?rhood OR revitali#ation adj3 urban OR urban health OR housing adj3 improvement OR home adj3 improvement OR affordable housing OR affordable home* OR housing intervention OR education OR whole school OR school adj3 environment OR greenspace or local government OR local authorit* OR council OR Public health/ OR Population Health/ OR Mental health/ OR wellbeing/ OR health disparity/ OR Health promotion/ OR welfare/ OR Social Determinants of Health/ OR Housing/ OR Crime/ OR Violence/ OR city planning/ OR urban health/ OR School/ OR Education/ OR Local government/ /Limit to English language 1,138,040

2) Knowledge exchange OR knowledge transfer OR knowledge translation OR knowledge dissemination OR knowledge diffusion OR knowledge utili#ation OR knowledge management OR knowledge adoption OR knowledge uptake OR evidence utili#ation OR “evidence use” OR “knowledge use” OR “research evidence” OR Knowledge management/ Limit to English language 17,993

3) Systematic review OR literature review OR evidence review OR narrative review OR scoping review OR critical review OR rapid review OR umbrella review OR “review of reviews” OR meta-analysis OR meta-ethnography OR Systematic review/ OR Meta-Analysis/ /Limit to English language 591,538

Scopus: 1 AND 2 AND 3 = 1,216 (title, abstract, keyword)

1) “Public health” OR “population health” OR “community health” OR “health inequalit*” OR “health inequit*” OR “well being” OR “place based” OR “community empowerment” OR “community transformation” OR “community asset*” OR “asset-based approach*” OR “upstream determinant*” OR “social determinant*” OR “health promotion” OR welfare OR benefits OR crime OR “community safety” OR transport OR housing OR planning W/3 town OR planning W/3 city OR planning W/3 neighborhood OR planning W/3 urban OR renewal W/3 town OR renewal W/3 city OR renewal W/3 neighborhood OR renewal W/3 urban OR development W/3 town OR development W/3 city OR development W/3 neighborhood OR development W/3 urban OR regeneration W/3 town OR regeneration W/3 city OR regeneration W/3 neighborhood OR regeneration W/3 urban OR revitali?ation W/3 town OR revitali?ation W/3 city OR revitali?ation W/3 neighbo?rhood OR revitali?ation W/3 urban OR “urban health” OR housing W/3 improvement OR home W/3 improvement OR “affordable housing” OR “affordable home*” OR “housing intervention” OR education OR “whole school” OR school W/3 environment OR greenspace or “local government” OR “local authorit*” OR council Limit to English 4,696,290

2) “Knowledge exchange” OR “knowledge transfer” OR “knowledge translation” OR “knowledge dissemination” OR “knowledge diffusion” OR “knowledge utili?ation” OR “knowledge management” OR “knowledge adoption” OR “knowledge uptake” OR “evidence utili?ation” OR “evidence use” OR “knowledge use” OR “research evidence” Limit to English language 101,760

3) “Systematic review” OR “literature review” OR “evidence review” OR “narrative review” OR “scoping review” OR “critical review” OR “rapid review” OR “umbrella review” OR “review of reviews” OR “meta-analysis” OR “meta-ethnography” Limit to English language 635,785

( ( TITLE-ABS-KEY ( "public health" OR "population health" OR "community health" OR "health inequalit*" OR "health inequit*" OR "well being" OR "place based" OR "community empowerment" OR "community transformation" OR "community asset*" OR "asset-based approach*" ) OR TITLE-ABS-KEY ( "social determinant*" OR "health promotion" OR welfare OR benefits OR crime OR "community safety" OR transport OR housing ) OR TITLE-ABS-KEY ( planning W/3 town OR planning W/3 city OR planning W/3 neighborhood OR planning W/3 urban OR renewal W/3 town OR renewal W/3 city OR renewal W/3 neighborhood OR renewal W/3 urban OR development W/3 town OR development W/3 city ) OR TITLE-ABS-KEY ( development W/3 neighborhood OR development W/3 urban OR regeneration W/3 town OR regeneration W/3 city OR regeneration W/3 neighborhood OR regeneration W/3 urban OR revitali?ation W/3 town OR revitali?ation W/3 city OR revitali?ation W/3 neighbo?rhood ) OR TITLE-ABS-KEY ( revitali?ation W/3 urban OR "urban health" OR housing W/3 improvement OR home W/3 improvement OR "affordable housing" OR "affordable home*" OR "housing intervention" OR education OR "whole school" OR school W/3 environment OR greenspace ) OR TITLE-ABS-KEY ( "local government" OR "local authorit*" OR council ) ) ) AND ( TITLE-ABS-KEY ( "Knowledge exchange" OR "knowledge transfer" OR "knowledge translation" OR "knowledge dissemination" OR "knowledge diffusion" OR "knowledge utili?ation" OR "knowledge management" OR "knowledge adoption" OR "knowledge uptake" OR "evidence utili?ation" OR "research evidence" ) OR TITLE-ABS-KEY ( "evidence use" OR "knowledge use" ) ) AND ( TITLE-ABS-KEY ( "Systematic review" OR "literature review" OR "evidence review" OR "narrative review" OR "scoping review" OR "critical review" OR "rapid review" OR "umbrella review" OR "review of reviews" OR "meta-analysis" OR "meta-ethnography" ) ) AND ( LIMIT-TO ( LANGUAGE , "English" ) )

Web of Science: 1 AND 2 AND 3 = 1,062 (topic search – includes title, abstract, keywords)

1) “Public health” OR “population health” OR “community health” OR “health inequalit*” OR “health inequit*” OR “well-being” OR wellbeing OR “place based” OR “community empowerment” OR “community transformation” OR “community asset*” OR “asset-based approach*” OR “upstream determinant*” OR “social determinant*” OR “health promotion” OR welfare OR benefits OR crime OR “community safety” OR transport OR housing OR planning NEAR/3 town OR planning NEAR/3 city OR planning NEAR/3 neighbo$rhood OR planning NEAR/3 urban OR renewal NEAR/3 town OR renewal NEAR/3 city OR renewal NEAR/3 neighbo$rhood OR renewal NEAR/3 urban OR development NEAR/3 town OR development NEAR/3 city OR development NEAR/3 neighbo$rhood OR development NEAR/3 urban OR regeneration NEAR/3 town OR regeneration NEAR/3 city OR regeneration NEAR/3 neighbo$rhood OR regeneration NEAR/3 urban OR revitali?ation NEAR/3 town OR revitali?ation NEAR/3 city OR revitali?ation NEAR/3 neighbo?rhood OR revitali?ation NEAR/3 urban OR “urban health” OR housing NEAR/3 improvement OR home NEAR/3 improvement OR “affordable housing” OR “affordable home*” OR “housing intervention” OR education OR “whole school” OR school NEAR/3 environment OR greenspace or “local government” OR “local authorit*” OR council Limit to English language 4,116,370

2) “Knowledge exchange” OR “knowledge transfer” OR “knowledge translation” OR “knowledge dissemination” OR “knowledge diffusion” OR “knowledge utili?ation” OR “knowledge management” OR “knowledge adoption” OR “knowledge uptake” OR “evidence utili?ation” OR “evidence use” OR “knowledge use” OR “research evidence” Limit to English language 50,020

3) “Systematic review” OR “literature review” OR “evidence review” OR “narrative review” OR “scoping review” OR “critical review” OR “rapid review” OR “umbrella review” OR “review of reviews” OR “meta-analysis” OR “meta-ethnography” Limit to English language 451,061

(TS=("Public health" OR "population health" OR "community health" OR "health inequalit*" OR "health inequit*" OR "well-being" OR wellbeing OR "place based" OR "community empowerment" OR "community transformation" OR "community asset*" OR "asset-based approach*" OR "upstream determinant*" OR "social determinant*" OR "health promotion" OR welfare OR benefits OR crime OR "community safety" OR transport OR housing OR planning NEAR/3 town OR planning NEAR/3 city OR planning NEAR/3 neighbo$rhood OR planning NEAR/3 urban OR renewal NEAR/3 town OR renewal NEAR/3 city OR renewal NEAR/3 neighbo$rhood OR renewal NEAR/3 urban OR development NEAR/3 town OR development NEAR/3 city OR development NEAR/3 neighbo$rhood OR development NEAR/3 urban OR regeneration NEAR/3 town OR regeneration NEAR/3 city OR regeneration NEAR/3 neighbo$rhood OR regeneration NEAR/3 urban OR revitali?ation NEAR/3 town OR revitali?ation NEAR/3 city OR revitali?ation NEAR/3 neighbo?rhood OR revitali?ation NEAR/3 urban OR "urban health" OR housing NEAR/3 improvement OR home NEAR/3 improvement OR "affordable housing" OR "affordable home*" OR "housing intervention" OR education OR "whole school" OR school NEAR/3 environment OR greenspace or "local government" OR "local authorit*" OR council) AND TS=("Knowledge exchange" OR "knowledge transfer" OR "knowledge translation" OR "knowledge dissemination" OR "knowledge diffusion" OR "knowledge utili?ation" OR "knowledge management" OR "knowledge adoption" OR "knowledge uptake" OR "evidence utili?ation" OR "evidence use" OR "knowledge use" OR "research evidence") AND TS=("Systematic review" OR "literature review" OR "evidence review" OR "narrative review" OR "scoping review" OR "critical review" OR "rapid review" OR "umbrella review" OR "review of reviews" OR "meta-analysis" OR "meta-ethnography")) AND LANGUAGE: (English)

Indexes=SCI-EXPANDED, SSCI, A&HCI, CPCI-S, CPCI-SSH, ESCI Timespan=1970-2020

Total identified through database searches: 2902

Duplicates identified in EndNote: 983

Duplicates identified in Covidence: 5

Total to screen: 1914
