# Supplementary material for: Strategies for knowledge exchange for action to address place-based determinants of health inequalities: an umbrella review
Source: J Public Health (Oxf). 2022 Nov 30;45(3):e467–77. doi: 10.1093/pubmed/fdac146 (PMC10470361; doi:10.1093/pubmed/fdac146)
Supplement: Supplementary_file_2_-_Data_extraction_form_fdac146 [file supplementary_file_2_-_data_extraction_form_fdac146.docx]

**Supplementary file 2: Data extraction form (updated 29 Nov 21) (adapted from McGowan et al, 2021^[[1]](#footnote-15831)^)**

| **Bibliographical** **details** | | | | | | |
| --- | --- | --- | --- | --- | --- | --- |
| **Author** |  | | | | | |
| **Year** |  | | | | | |
| **Title** |  | | | | | |
| **Name of data extractor** | |  | | | **Date** |  |
|  | | | | | | |
| **Review details – include page numbers for extracted information** | | | | | | |
| **Research question addressed by systematic review** | | | |  | | |
| **Explicit interest in place-based determinants of health**  If yes, provide place-based determinant(s) with n of studies for each | | | |  | | |
| **Population**  Any age/gender/location etc. | | | |  | | |
| **Definition of knowledge exchange or related terms (e.g. knowledge translation/ transfer/management, dissemination) used by review**  Clear, succinct details should be provided, including KE model underpinning theory, type of knowledge, target audience. | | | | **Definition (explicit or implicit):**  **Model:**  **Theory:**  **Knowledge type:**  **Target audience:** | | |
| **Setting/context**  Physical, social, or economic environment and country/region | | | |  | | |
| **Health/health inequalities outcome(s)**  Included in search strategy  Reported in results | | | |  | | |
| **Sub-group analysis undertaken**  Describe population groups using PROGRESS+ Factors.  **(**Place of residence; Race/ ethnicity/ culture/ language; Occupation; Gender/ sex; Religion; Education; SES; Social capital; personal characteristics associated with discrimination (e.g. age, disability); features of relationships (e.g. smoking parents, excluded from school; time-dependent relationships (e.g. leaving the hospital, respite care, other instances where a person may be temporarily at a disadvantage) | | | |  | | |
| **Data sources searched**  Number of databases searched and/or other search strategies | | | |  | | |
| **Time limits of database search** | | | |  | | |
| **Number of studies in review (total & relevant)**  Base on review flow chart/ summary characteristic table or inclusion criteria if possible  Relevant studies = those reporting knowledge exchange and place-based determinants of health | | | | **Total:**  **Relevant:** | | |
| **Date range of included studies (total & relevant)**  The date range spanning from the earliest study that informs the included research synthesis to the latest should be reported. If this is not readily identifiable in the table of study characteristics provided by the included synthesis, it should be discernible by scanning the date range of publications through the results section of the included review. **Can report full date range but only extract from relevant studies interventions.** | | | | **Total:**  **Relevant:** | | |
| **Study design of included studies (include frequency)**  e.g. RCTS, Non-randomised controlled trials, controlled prospective cohort, repeat cross sections, natural experiments, observational studies etc. | | | | **Total:**  **Relevant:** | | |
| **Method of synthesis**  The type of research synthesis as stated by the authors of the included review should be detailed e.g. random effects meta-analysis, fixed effect meta-analysis, meta- aggregative synthesis, meta-ethnography, or narrative synthesis. | | | |  | | |
| **Quality** (as measured by systematic review authors)  The instrument or tool used to assess risk of bias, rigor or study quality should be reported along with some summary estimate of the quality of primary studies in the included research synthesis. | | | |  | | |
| **Key review findings**  Copy and paste from abstract | | | |  | | |
| **Key review conclusions**  Copy and paste from abstract | | | |  | | |
| **Are all included studies relevant for this umbrella review?** | | |  | If Y, go to ‘Summary table’; if N, complete ‘Primary studies findings’ | | |

**SUMMARY TABLE**

- If more than one type of KE included in the review, consider grouping the extracted data by KE model type or between different setting/place-based determinant

| **Summary table** | | | | | | | |  |
| --- | --- | --- | --- | --- | --- | --- | --- | --- |
| ***Review*** | ***Context (setting^[[2]](#footnote-1)^, country)*** | ***Intervention(s)*** *If more than 1 intervention, enter n of studies*  *KE Model (evidence access, active KE or both)*  *Theories underpinning KE model*  *Knowledge type (research evidence/data/lay/policy/practitioner/*  *formalized knowledge)*  *Target audience, if stated (e.g. practitioner/policy, community/public)*  *KE activity description* | ***Key findings***  *Copy from discussion intro*  *List result section headings if helpful..* | ***Effectiveness/outcomes of KE***  *(e.g. negative, positive, unintended outcomes)* | ***PROGRESS +***  *(leave in for piloting)* | ***KE Context***   1. *Barriers to KE* 2. *Facilitators to KE* | ***Impact on health/health inequalities outcome(s)***   1. *Does model of KE aim to address upstream/structural determinants of inequalities* 2. *Does the model of KE promote equity focus (e.g. is model of KE inclusive of lived lay experiences of inequalities, challenging power etc.)* 3. *Are health inequalities outcomes reported including differential impacts (e.g. Progress factors* | **Any other Recommendations for KE** |
|  |  |  |  |  |  |  |  |  |

**PRIMARY STUDIES**

- If there fewer than five studies in a review that are relevant, these can be included individually in full here.
- If there are more than five, studies should be grouped by similar interventions. Define similar interventions based on their approach to KE (e.g. evidence access versus active KE)

| **Primary studies’ findings - Part 1 (Study characteristics)** | | | | | |
| --- | --- | --- | --- | --- | --- |
| For relevant studies in the review summarise: | | | | | |
| ***Systematic***  ***review author (year)*** | ***Author(s) of original publication***  ***Year of publication***  ***Relevant page numbers*** | ***Study design*** | ***Study aim/ research question*** | ***Setting and participants***  ***(Country, region, organisational setting, place-based determinant, sample size)*** | ***Quality appraisal***  ***(as assessed by systematic review authors*** |
|  |  |  |  |  |  |
|  |  |  |  |  |  |
|  |  |  |  |  |  |
|  |  |  |  |  |  |

| **Primary studies’ findings - Part 2 (KE description and findings)** | | | | | | | | |
| --- | --- | --- | --- | --- | --- | --- | --- | --- |
| For relevant studies in the review summarise: | | | | | | | | |
| ***System.***  ***Review author*** | ***Author(s) of original publication****^^[[3]](#footnote-2)^^*  ***Relevant page numbers/ appendices*** | ***Intervention description***   1. *KE Model (evidence access, active KE or both)* 2. *Theories underpinning KE model* 3. *Knowledge type (research evidence/data/lay/policy/practitioner/*   *formalized knowledge)*   1. *Target audience, if stated (practitioner/policy, community/public)* 2. *KE activity description* | ***Key findings***  *As described in review* | ***Effectiveness/outcomes of KE***  *(e.g. negative, positive, unintended outcomes)* | ***Findings WRT PROGRESS +*** | ***KE Context***   1. *Barriers to KE* 2. *Facilitators to KE* | ***Impact on health/health inequalities outcome(s)***   1. *Does model of KE aim to address upstream/structural determinants of inequalities* 2. *Does the model of KE promote equity focus (e.g. is model of KE inclusive of lived lay experiences of inequalities, challenging power etc.)* 3. *Are health inequalities outcomes reported including differential impacts (e.g. Progress factors* | ***Any other author recommendations for KE not already described.*** |
|  |  |  |  |  |  |  |  |  |
|  |  |  |  |  |  |  |  |  |
|  |  |  |  |  |  |  |  |  |

1. McGowan VJ, Buckner S, Mead R, McGill E, Ronzi S, Beyer F, Bambra C. Examining the effectiveness of place-based interventions to improve public health and reduce health inequalities: an umbrella review. BMC Public Health. 2021 Oct 19;21(1):1888. doi: 10.1186/s12889-021-11852-z. PMID: 34666742; PMCID: PMC8524206. [↑](#footnote-ref-15831)
2. Setting also includes place-based factors – social, physical or economic environment – in addition to country/region [↑](#footnote-ref-1)
3. If there fewer than five studies that are relevant, these can be included in full here. However, if there are more than five, studies should be grouped if interventions are similar. Define similar interventions based on their approach to KE – Evidence access versus active KE [↑](#footnote-ref-2)
