# Supplementary material for: Strategies for knowledge exchange for action to address place-based determinants of health inequalities: an umbrella review
Source: J Public Health (Oxf). 2022 Nov 30;45(3):e467–77. doi: 10.1093/pubmed/fdac146 (PMC10470361; doi:10.1093/pubmed/fdac146)
Supplement: Supplementary_file_3a_-_Review_Characteristics_fdac146 [file supplementary_file_3a_-_review_characteristics_fdac146.docx]

**Supplementary file 3a: Review Characteristics**

| **Review: Author (Year)** | **Review aims/questions** | **Full or partial relevance** | **Review design (methods of synthesis)** | **Place based determinant(s) number of relevant studies** | **Population** | **Country/ies and region** | **Setting** | **Time limits of search (Data sources searched)** | **Number of studies in review: total (relevant)** | **Quality appraisal (as assessed by authors)** |
| --- | --- | --- | --- | --- | --- | --- | --- | --- | --- | --- |
| Armstrong (2013)^(1)^ | To understand potential barriers and facilitators, types of knowledge translation (KT) strategies and theoretical perspectives described and applied in contexts relevant to public health decision-making; To assess the effectiveness of KT interventions for public health decision makers and managers | Partial | Not reported | Relevant studies consider the social determinants of health/public health including waste management, animal management, recreation and culture | Local government decision makers | Not prespecified by review (relevant study Canada) | Local government | Not reported (Medline, CINAHL, APAIS, PsychInfo, Web of Science, Google, and Google Scholar including peer reviewed and grey literature) | 16 (1) | Not undertaken |
| Cohen (2017)^(2)^ | To obtain an overview of the literature related to public health advocacy, with a particular interest in the extent to which this literature addresses the goal of reducing the social, environmental and structural causes of health and social inequities. | Partial | A scoping review (thematic analysis) | Relevant studies consider food insecurity, transportation, tobacco control, health inequalities and the social determinants of health | Not specified | Not prespecified by review (relevant studies: USA (5); Canada (1); Australia) | Population or community settings | 2000-2015 (Databases (PubMed, CINAHL, PsycINFO and Social Sciences Citation Index)  Selected articles were used for citation snowballing Grey literature databases ( Google Scholar, Google, Google Books, ProQuest Dissertations and Theses, Grey Literature Report) Relevant organisational websites and library catalogues of Canadian universities) | 183 studies (8*)  *Includes one review Farrer (2015) extracted in own right | Not undertaken |
| Farrar (2015)^(3)^ | To synthesize the evidence in the academic and gray literature and to provide a body of knowledge for advocates to draw on to inform their advocacy efforts that seek to promote policies that improve health equity by challenging unequal exposure to—and distributions of—the social determinants of health | Partial | A synthesis review (critical interpretive synthesis) | Relevant studies consider the social determinants of health including employment and working conditions, income, and social protection (including poverty, discrimination, and disadvantage). | Decision makers and policy makers in general | Not prespecified by review (relevant studies: Australia, US, Canada (=3), international) | Local and national government settings | Jan 1990 - Mar 2013 (PubMed, Web of Science, PsycINFO (EBSCO), and SocInfo ; gray literature repositories and internet searches) | Academic literature 137 plus grey literature 59 (7) | Not undertaken |
| Haynes (2018)^(4)^ | What causal mechanisms can best explain the observed outcomes of interventions that aim to increase policy-makers’ capacity to use research in their work? | Partial | A exploratory realist scoping review (realist interpretation) | Relevant study considers 'healthy cities' | Policy makers | Not prespecified by review (included study the Netherlands) | Local and national government, health service settings | 2001-2016 (3 published reviews, PAIS and Web of Science databases, iterative searches on Google and Google Scholar and snowballing from citations in previously found papers, reports and journal articles) | 22 (1) | Based on the appraisal criteria used in critical interpretive synthesis, studies were excluded if they were “fatally flawed”. |
| Jakobsen (2019)^(5)^ | To review knowledge on organisational factors that facilitate research use in public health policy-making, | Partial | A scoping review (thematic content analysis) | relevant study considers the built environment | Policy officials and policy makers | Not prespecified by review (included study the Netherlands) | Public policy making settings | 1970- July 2017 (PubMed, Academic Search Premier and Scopus; manual screening of document repositories on institutional websites) | 54 (1) | Authors note that quality appraisal not undertaken because this was a scoping review |
| Kneale (2017)^(6)^ | To map the use of research evidence in public health decision-making at a sub-national level, and where possible to compare patterns of evidence utilisation before the reconfiguration of public health services (2010–2012/13) and afterwards (2013/14–2016). | Partial | A systematic scoping review (narrative, configurative approach to synthesis) | Relevant studies have a general place based interest; consider alcohol licensing, housing, leisure and recreation, planning, construction and health inequity | Public health decision makers and professionals | Reporting findings from English public health systems (relevant studies: England, Scotland Wales (1); Engand (3); England and International (1), UK (2) | Public health settings | 2010-2016 (Databases (PubMed, HMIC, EconLit and Scopus); manual searching and itation tracking) | 23 papers from 21 studies (7) | Not undertaken |
| Lorenc (2014)^(7)^ | How do local decision-makers in policy sectors broadly related to the built environment use and/or perceive research evidence | Full | A systematic review of qualitative evidence (thematic synthesis) | The built environement | Policy-makers, practitioners or anyone with a local-level decision-making role | High income countries | Local policy settings | Not reported (28 databases, 5 key journals, reference searching of included studies) | 16 (16) | Majority of studies rated as low or medium quality |
| Masood (2020)^(8)^ | The primary objective was to systematically examine studies exploring the use of research evidence in public health policy decision making (updating Orton, 2011 review) | Partial | A systematic review (narrative synthesis) | Relevant studies have a general place based interest | Public health decision makers | Countries with universal healthcare coverage (relevant studies: Australia (1); Uk (1) | Public health settings | 2010- Jan 2016 (14 electronic databases; websites of key organizations; reverse citation search; forward citation search; internet search engines such as Google and Google Scholar | 16 (2) | Critical Appraisal Skills Programme (CASP) and Mixed Methods Appraisal Tool (MMAT) |
| McDonald (2016)^(9)^ | To examine the extent, range and nature of literature about health-related knowledge transfer in Inuit communities. | Partial | A scoping review (charting and thematic analysis) | Relevant studies consider environmental health and food production | Inuit populations | Circumpolar locations (relevant studies: Northern Canada (3) | Circumpolar locations | Databases any year (Proquest; EBSCOHost) Handsearched key journals in field | 39 (3) | Not undertaken |
| Oliver (2014)^(10)^ | To identify factors which act as barriers to and facilitators of the use of evidence in public policy, including factors perceived by different stakeholder groups;To describe the focus, methods, populations, and findings of the new evidence in this area | Partial | A systematic review of barriers and faciliators (coding of barriers and facilitators) | Relevant studies have a general place based interest; consider health inequalities; general social determinants of health; employment/ income specifically; environmental health; landscape quality; and smoke free places. | Public policy makers | Not prespecified by review (relevant studies: Canada (=3), Netherlands (=2), UK (=2), Australia, USA, International | State organisation, or a group of state organisations, at a national, regional or conurbation level. | Jul 2000 - Sept 2012 (Medline, Embase, SocSci Abstracts, CDS, DARE, Psychlit, Cochrane Library, NHSEED, HTA, PAIS, IBSS. Authors in the field were contacted and key websites were hand-searched) | 145 (10) | Not undertaken |
| Orton (2011)^(11)^ | To synthesise empirical evidence on the use of research evidence by public health decision makers in settings with universal health care systems | Partial | A systematic review (narrative synthesis) | Fields that impact that health including fiscal, agricultural, transport, town planning and crime | Public health decision makers | Countries with universal health care systems | Universal health care systems | 1980- Mar 2010 (MEDLINE, SCOPUS, PsychInfo, CINAHL, The Social Science Citation Index, The Science Citation Index, The Arts and Humanities Citation Index, Applied Social Sciences Index and Abstracts (ASSIA), Database of Reviews of Effects (DARE), Cochrane Database of Systematic Reviews (CDSR), DoPHER, the Campbell Library, and the Cochrane Register of Controlled trials (CENTRAL). General internet search engines and websites of key organisations were scanned. Colleagues and key organisations were also contacted and the reference lists of all included studies were scrutinised) | 18 (4) | Quality was mixed based on relevent CASP tool. Overall rating for each study not reported. |
| Plamondon (2019)^(12)^ | What promising practices for connecting knowledge with action (KWA) for health equity are evident in the literature? | Partial | A critical interpretive synthesis (iterative qualitative analysis and synthesis) | Relevant studies consider health equity, the social determinants or health and environmrnental health. | Not specified | Not prespecified by review (relevant studies: Canada (3); Australia (2); USA (2) | Health care and public health settings | 2010-2016 (Literature identified from previously published scoping review. Not reported but reference provided) | 32 (7) | Used criteria developed by Dixon-Woods et al. (2006). All studies reported as relatively high quality. |
| Salsberg (2015)^(13)^ | To describe key strategies supporting development of participatory research (PR) teams to engage partners for creation and translation of action-oriented knowledge | Partial | A critical review (deductive qualitative thematic analysis) | relevant studies consider the social determinants of health or environmental health | Partners in participatory research | North America | Community settings | 1995- October 2009 (The research of four leading participatory researcher practitioners identified via their Citespace centrality score. Searches was conducted to identify their published materials in PubMed, Embase, ISI Web of Science, PsychInfo, and CAB (Ovid database). Chapters from books they edited were also considered). | 54 (7) | Not undertaken |
| Wine (2017)^(14)^ | To explore and identify the extent and nature of the scholarly literature regarding research studies that address or describe experiences or research on the collaborative research processes in the context of environmental health research.  Guiding question: What are the specific components that influence the collaborative research process in environmental health research? | Full | A scoping review (charting and thematic analysis, engaged stakeholders) | Environmental health | Not specified | Not prespecified by review (relevant studies: (USA (35); Canada (4); New Zealand (2); Italy; Sweden; Netherlands; UK (1) | Environmental health contexts | Any time until August 2015 (Databases (Medline (Ovid), EMBASE (Ovid), CINAHL(EBSCO), Global Health (Ovid), SocINDEX (EBSCO), Scopus, Pollution Abstracts (ProQuest), Environment Complete (EBSCO), ProQuest Dissertations, and Theses Global) Additional references identified through citations or other known publications) | 45 | Authors note that quality appraisal not undertaken because this was a scoping review |

**Included review references**

1. Armstrong R, Waters E, Dobbins M, Anderson L, Moore L, Petticrew M, et al. Knowledge translation strategies to improve the use of evidence in public health decision making in local government: intervention design and implementation plan. Implementation Science. 2013; 8:121. 10.1186/1748-5908-8-121.

2. Cohen BE, Marshall SG. Does public health advocacy seek to redress health inequities? A scoping review. Health & Social Care in the Community. 2017; 25:309-28. doi.org/10.1111/hsc.12320

3. Farrier L, Marinetti C, Cavaco YK, Costongs C. Advocacy for Health Equity: A Synthesis Review. The Milbank Quarterly. 2015; 93:392-437. doi.org/10.1111/1468-0009.12112.

4. Haynes A, Rowbotham SJ, Redman S, Brennan S, Williamson A, Moore G. What can we learn from interventions that aim to increase policy-makers’ capacity to use research? A realist scoping review. Health research policy and systems. 2018; 16:1-27.

5. Jakobsen MW, Eklund Karlsson L, Skovgaard T, Aro AR. Organisational factors that facilitate research use in public health policy-making: a scoping review. Health Research Policy and Systems. 2019; 17:90. 10.1186/s12961-019-0490-6.

6. Kneale D, Rojas-García A, Raine R, Thomas J. The use of evidence in English local public health decision-making: a systematic scoping review. Implementation Science. 2017; 12:53. doi.org/10.1186/s13012-017-0577-9.

7. Lorenc T, Tyner EF, Petticrew M, Duffy S, Martineau FP, Phillips G, et al. Cultures of evidence across policy sectors: systematic review of qualitative evidence. The European Journal of Public Health. 2014; 24:1041-7.

8. Masood S, Kothari A, Regan S. The use of research in public health policy: a systematic review. Evidence & Policy: A Journal of Research, Debate and Practice. 2020; 16:7-43.

9. McDonald ME, Papadopoulos A, Edge VL, Ford J, Sumner A, Harper SL. What do we know about health-related knowledge translation in the Circumpolar North? Results from a scoping review. International Journal of Circumpolar Health. 2016; 75:31223. 10.3402/ijch.v75.31223.

10. Oliver K, Innvar S, Lorenc T, Woodman J, Thomas J. A systematic review of barriers to and facilitators of the use of evidence by policymakers. BMC Health Services Research. 2014; 14:2. doi.org/10.1186/1472-6963-14-2.

11. Orton L, Lloyd-Williams F, Taylor-Robinson D, O'Flaherty M, Capewell S. The Use of Research Evidence in Public Health Decision Making Processes: Systematic Review. PLOS ONE. 2011; 6:e21704. doi.org/10.1371/journal.pone.0021704.

12. Plamondon KM, Caxaj CS, Graham ID, Bottorff JL. Connecting knowledge with action for health equity: a critical interpretive synthesis of promising practices. International Journal for Equity in Health. 2019; 18:202. doi.org/10.1186/s12939-019-1108-x.

13. Salsberg J, Parry D, Pluye P, Macridis S, Herbert CP, Macaulay AC. Successful Strategies to Engage Research Partners for Translating Evidence into Action in Community Health: A Critical Review. Journal of Environmental and Public Health. 2015; 2015:191856. 10.1155/2015/191856.

14. Wine O, Ambrose S, Campbell S, Villeneuve PJ, Burns KK, Vargas AO. Key Components of Collaborative Research in the Context of Environmental Health: A Scoping Review. Journal of Research Practice. 2017; 13:R2.
