# Supplementary material for: Strategies for knowledge exchange for action to address place-based determinants of health inequalities: an umbrella review
Source: J Public Health (Oxf). 2022 Nov 30;45(3):e467–77. doi: 10.1093/pubmed/fdac146 (PMC10470361; doi:10.1093/pubmed/fdac146)
Supplement: Supplementary_file_4_-_CASP_findings_fdac146 [file supplementary_file_4_-_casp_findings_fdac146.docx]

**Supplementary fine 4: Findings from the Critical Appraisal Skills Programme Systematic Review Checklist – Items 1 to 5**

Reference: Critical Appraisal Skills Programme (2018). CASP Systematic Review Checklist. [online] Available at: https://casp-uk.b-cdn.net/wp-content/uploads/2021/11/CASP-Systematic-Review-Checklist_2018_word.docx. Accessed: Date Accessed 14 Mar 2022

| **Review lead author (year)** | **1. Did the review address a clearly focused question?** | **2. Did the authors look for the right type of papers?** | **3. Do you think all the important, relevant studies were included?** | **4. Did the review’s authors do enough to assess quality of the included studies?** | **5. If the results of the review have been combined, was it reasonable to do so?** |
| --- | --- | --- | --- | --- | --- |
| Armstrong et al. (2013) | Yes | Can't tell | Can't tell | No | Can't tell |
|  | Scoping review aim: To understand potential barriers and facilitators, types of knowledge strategies and theoretical perspectives described and applied in contexts relevant to public health decision making. | It was not possible to report the extensive methodology in the review article. | It was not possible to report the extensive methodology in the review article. | No quality assessment undertaken, no rationale given. | It was not possible to report the extensive methodology in the review article. |
| Cohen and Marshall (2017) | Yes | Yes | Yes | No | Yes |
|  | To obtain an overview of the literature related to public health advocacy, with a particular interest in the extent to which this literature addresses the goal of reducing the social, environmental and structural causes of health and social inequities. | Review looked for studies of advocacy involving population or community-level advocacy initiatives. | Databases searched. Selected articles were used for citation snowballing. Grey literature databases, relevant organisational websites and library catalogues of Canadian universities. | Not quality assessment undertaken, no rationale given. | Thematic analysis was both inductive and deductive according to the objectives. |
| Farrer et al. (2015) | Yes | Yes | Yes | No | Yes |
|  | What evidence exists in the academic and gray literature about effective advocacy for health equity? | No limits on study design applied. | Four electronic databases were for cross-disciplinary coverage: Also searched gray literature using Internet-based repositories and consulted experts in the field. | No quality assessment undertaken, no rationale given. | No formal meta-analysis undertaken. Papers coded using dimensions of advocacy for health equity. |
| Haynes et al. (2018) | Yes | Yes | Yes | Yes | Yes |
|  | What causal mechanisms can best explain the observed outcomes of interventions that aim to increase policy-makers’ capacity to use research in their work? | Evaluations including process evaluations and reports of ‘soft’ proximal outcomes such as satisfaction and awareness. | Included references of three papers that evaluated strategies aimed at increasing the research use; Two searches on academic databases (PAIS and Web of Science) searching between 2001 and 2016; Key word and citation searches on Google and Google Scholar. | Studies were excluded if they were “fatally flawed” criteria as per Dixon Woods' appraisal for critical interpretive synthesis. A low threshold for quality was set due to the diversity of methodologies and because data synthesis would include judgements about the credibility and contribution of studies. | Using a realist approach, articles were reviewed for information about contexts, outcomes (including process effects) and possible causal mechanisms. |
| Jakobsen et al. (2019) | Yes | Yes | Yes | Yes | Yes |
|  | To create add-on knowledge to existing frameworks for understanding research use in policy organisations and to identify possible research gaps. | Studies are not limited by methods. | Multiple multidisciplinary database searches. Screened document repositories on institutional websites known to have contributed to the knowledge base; screened reference lists of included studies. | Authors did not conduct a quality assessment explaining it as the norm for this type of review. | Thematic content analysis. Due to the large number of included studies (n=54) from a wide range of settings, limited amounts of information from individual studies could be reported. |
| Kneale et al. (2017) | Yes | Yes | Yes | Yes | Yes |
|  | To map the use of research evidence in public health decision-making at a sub-national level, and where possible to compare patterns of evidence utilisation before the reconfiguration of public health services (2010–2012/13) and afterwards (2013/14–2016) | Broad set of inclusion criteria related to study design.. | Range of databases searched, including manually. No reference to personal contact with experts or inclusion of unpublished studies. | Formal quality appraisal not undertaken of individual studies as review sought to map available literature and undertake groundwork for future primary research. | Narrative, configurative approach to synthesis. |
| Lorenc et al. (2014) | Yes | Yes | Yes | Yes | Yes |
|  | How do local decision-makers in policy sectors broadly related to the built environment use and/or perceive research evidence? Defines research evidence as that produced by academics; in high income countries. | Focused on qualitative data to gain a more nuanced picture of cultures of evidence in their practical context. | Three strategies used (databases searched n=28; handsearching and citation searches). Authors did not include/search for grey/ unpublished studies, however, the focus of review was on academic research only. | The quality appraisal tool produced by the Centre for Public Health Excellence at the UK National Institute for Health and Care Excellence used. | Thematic synthesis. Some discussion of differing findings by sector. |

| **Review lead author (year)** | **1. Did the review address a clearly focused question?** | **2. Did the authors look for the right type of papers?** | **3. Do you think all the important, relevant studies were included?** | **4. Did the review’s authors do enough to assess quality of the included studies?** | **5. If the results of the review have been combined, was it reasonable to do so?** |
| --- | --- | --- | --- | --- | --- |
| Masood et al. (2020) | Yes | Yes | Yes | Yes | Yes |
|  | The primary objective was to systematically examine studies exploring the use of research evidence in public health policy decision making (updating Orton, 2011 review) | Studies must provide empirical data, but can be of any design. | Searched several databases, internet sources and forward/backward citation used. | Critical Appraisal Skills Programme (CASP) used for qualitative and quantitative studies. Mixed Methods Appraisal Tool (MMAT) was used for mixed-methods studies. | Narrative synthesis bringing both qualitative and quantitative evidence together to deduce findings and interpretations. |
| McDonald et al. (2016) | Yes | Yes | Yes | No | Yes |
|  | To examine the extent, range and nature of literature about health-related knowledge in Inuit communities. | Broad inclusion criteria related to study design for searches. | Included theses and grey literature as well as peer reviewed journal articles. Identified studies relevant to Inuit populations in Circumpolar locations | No quality assessment undertaken. No rationale given. | Thematic analysis undertaken alongside charting of results (numerical and narrative form). |
| Oliver et al. (2014) | Yes | Yes | Yes | Yes | Yes |
|  | To identify factors which act as barriers to and facilitators of the use of evidence in public policy, including factors perceived by different stakeholder groups. | Primary research or systematic reviews; a range of study designs and theoretical papers. | Authors in the field were contacted and key websites were hand-searched plus multiple electronic databases searched. | No quality assessment undertaken. Rationale: Quality appraisal would have made no difference to this systematic descriptive synthesis of a body of literature. | Factors which affected evidence use were coded as barriers or facilitators against a pre-defined list of factors, which was iteratively updated as new factors were identified. |
| Orton et al. (2011) | Yes | Yes | Yes | Yes | Yes |
|  | To synthesise empirical evidence on the use of research evidence by public health decision makers in settings with universal health care systems. | Any study design was considered eligible, so long as it revealed empirical data relating to the review objectives. | 13 bibliographic databases were screened, organisational websites were scanned, key informants were contacted and bibliographies of included studies scanned. | Methodological quality was assessed using CASP tools to provide a qualitative assessment rather than rating the studies as high or low quality. | Narrative synthesis. |
| Plamondon et al. (2019) | Yes | Yes | Can't tell | Yes | Yes |
|  | What promising practices for connecting knowledge with action (KWA) for health equity are evident in the literature? | Explicit focus on KE in the search strategy. | Search strategies not reported in this article though authors cite an accompanying article where methods are reported. Limitations acknowledge that grey and published studies not included. | Applied criteria developed by Dixon Wood, and report quality score for individual studies | Critical interpretive synthesis undertaken. Supplementary tables detail different knowledge strategies, grouping these by individual studies. |
| Salsberg et al. (2015) | Yes | Yes | No | No | Can't tell |
|  | To undertake a critical review describing key strategies supporting development of participatory research (PR) teams to engage partners for creation and translation of action-oriented knowledge. | No limits on study design applied. | The review limited its scope to the work of 4 leading practitioners (all based in North America). | No quality assessment undertaken. No discussion of the quality of the evidence base. | A critical review: It is unclear which studies informed which findings. No discussion of how the findings might vary by setting. |
| Wine et al. (2017) | Yes | Yes | Yes | Yes | Yes |
|  | What are the specific components that influence the collaborative research process in environmental health research? | Broad inclusion criteria related to study design for searches. | Searched range of databases across disciplines. | No quality assessment undertaken. Rationale: because this is a scoping review | Charting and thematic synthesis undertaken across studies. |

**Supplementary information: Findings from the Critical Appraisal Skills Programme Systematic Review Checklist – Items 6 to 10.**

| **Review lead author (year)** | **6. What are the overall results of the review?** | **7. How precise are the results?** | **8. Can the results be applied to the local population?** | **9. Were all important outcomes considered?** | **10. Are the benefits worth the harms and costs?** |
| --- | --- | --- | --- | --- | --- |
| Armstrong et al. (2013) |  |  | Can't tell | Can't tell | Can't tell |
|  | The systematic and scoping reviews identified that effective and promising strategies to increase access to research evidence require an integrated intervention of skill development, access to a knowledge broker, resources and tools for evidence-informed decision making, and networking for information sharing. | Not applicable, qualitative themes. | No information provided about the settings/ population of the included studies. | Methods of synthesis not provided. | Very limited details reported. |
| Cohen and Marshall (2017) |  |  | Yes | Yes | Can't tell |
|  | Although public health advocacy to address root causes of health inequities is supported theoretically and through professional practice standards, the empirical literature does not reflect that this is occurring widely in public health practice. Tensions within the discourse were noted and multiple barriers to engaging in public health advocacy for health equity were identified, including a preoccupation with individual responsibilities for healthy lifestyles and behaviours, consistent with the emergence of neoliberal governance. | Not applicable, qualitative themes. | Recommendations for public health practice regarding future advocacy related to social determinants of health and health inequalities given. | Outcomes not predefined, some descriptive examples emerge through synthesis. | No mention of unintended consequences, negative effects or harms arising from advocacy. |
| Farrer et al. (2015) |  |  | Yes | Yes | Can't tell |
|  | The policy world is complex, and scientific evidence is unlikely to be conclusive in making decisions. The potential impact of evidence can be increased by “packaging” it as part of knowledge transfer and translation. Increased contact between researchers and policymakers could improve the uptake of research in policy processes. Researchers can play a role in advocacy efforts, although health professionals and disadvantaged people can be particularly persuasive in advocacy efforts. Several barriers hamper advocacy efforts. The most frequently cited in the academic literature are the current political and economic zeitgeist and related public opinion, which tend to blame disadvantaged people for their ill health, even though biomedical approaches to health and political short-termism also act as barriers. | Not applicable, qualitative themes. | Brings together evidence from the academic and the gray literature in how to advocate for health equity based on evidence largely from the English-speaking world | Predeveloped coding framework based on literature and expert opinion regarding the dimensions of health equity applied. | No mention of unintended consequences, negative effects or harms arising from advocacy. |
| Haynes et al. (2018) |  |  | Yes | Yes | Yes |
|  | Many potential mechanisms were identified as well as some enduring contextual characteristics that all interventions should consider. The evidence was variable, but the SCMO analysis suggested that tailored interactive workshops supported by goal-focused mentoring, and genuine collaboration, seem particularly promising. Systems supports and platforms for cross-sector collaboration are likely to play crucial roles. | Not applicable, qualitative themes. | Studies from low- and middle-income countries included. The authors argue that whilst these settings are likely to differ considerably, together, the studies provide insights into creative interventions and produce findings that may have implications across the country income divide. | An inductive approach to analysis guided by realist thinking rather than starting with an a priori framework or conceptual categories. | Realist approach considered intended and unintended consequences. |
| Jakobsen et al. (2019) |  |  | Can't tell | Yes | Can't tell |
|  | The synthesis shows a lack of studies on politicians and the need for more theoretically founded research. Despite increased efforts to update the existing evidential and theoretical basis of research use, we still need frameworks that combine different approaches and theories to help us grasp the complex organisational mechanisms that facilitate research use in policy settings. | Not applicable, qualitative themes. | The intra-organisational factors influencing research use in policy-making were systematically mapped and the theories applied in this research area were assessed. A mix of national, regional and local settings -limited discussion of how findings vary by setting. | Thematic content analysis to develop open codes of organisational factors identified in the study outcomes. | No mention of unintended consequences, negative effects or harms arising from advocacy. |

| **Review lead author (year)** | **6. What are the overall results of the review?** | **7. How precise are the results?** | **8. Can the results be applied to the local population?** | **9. Were all important outcomes considered?** | **10. Are the benefits worth the harms and costs?** |
| --- | --- | --- | --- | --- | --- |
| Kneale et al. (2017) |  |  | Yes | Yes | Can't tell |
|  | Three trends were identified: (i) the primacy of local evidence, (ii) the importance of local experts in providing evidence and knowledge, and (iii) the high value placed on local evaluation evidence despite the varying methodological rigour. Barriers included access and availability of applicable research evidence, and indications that evidence use could be seen as bureaucratic. Two new factors from public health structures reform (i) greater emphasis on the perceived uniqueness of LA areas following public health devolution (ii) challenges introduced following increased local political accountability. | Not applicable, qualitative themes. | Although authors note the 'narrow focus' on local public health decision-making in England could mean that the results have limited applicability to other settings, however, they contextualise findings with respect to evidence base more generally. | Evidence of impact emerged through synthesis - inductive coding and framework development. | No mention of unintended consequences, negative effects or harms arising from evidence-based decision-making. |
|  |  |  | Yes | Yes | Can't tell |

| Lorenc et al. (2014) | Several factors are seen to influence decision-makers’ views of evidence, including practical factors such as resources or organizational support; the credibility of the evidence; its relevance or applicability to practice; considerations of political support or feasibility; and legislative constraints. There are limited data on how evidence is used: it is sometimes used to not only support decision-making, but also to lend legitimacy to decisions that have already been made. | Not applicable, qualitative themes. | Identifies cultures of evidence for local policy makers concerned with the built environment and health based in higher income countries. | Outcomes not prespecified. Data were coded line-by-line, using an open code set, and synthesized thematically using constant comparison. | No mention of unintended consequences, negative effects or harms arising from evidence-based decision-making. |
| --- | --- | --- | --- | --- | --- |

| Masood et al. (2020) |  |  | Yes | Yes | Can't tell |
| --- | --- | --- | --- | --- | --- |
|  | Findings align with previous literature to show that various types of research evidence are being accessed in public health policymaking. Challenges and enablers exist at multiple levels of the system, suggesting that use of research evidence is a complex, interdependent process. | Not applicable, qualitative themes. | Findings can be used to determine how to engage both public health professionals and researchers in exploring processes of learning, negotiation and capacity building, so that the two communities can function effectively and efficiently separately and together. | Narrative synthesis, a critical analysis approach that deduces findings and interpretations. Approach informed by Orton et al (2011)'s review. | No mention of unintended consequences, negative effects or harms arising from evidence-based decision-making. |
| McDonald et al. (2016) |  |  | Yes | Yes | Yes |
|  | Analysis identified 3 themes: the value of community stakeholders as active members in the research process; the importance of local context in tailoring knowledge transfer strategies; and the challenges with varying and contradictory health messaging in knowledge transfer. A lack of critical assessment of community involvement in research and of knowledge transfer were identified. Assessments rarely focused on whether knowledge transfer had elicited its intended action. | Not applicable, qualitative themes. | Conclusions provide recommendations for future knowledge exchange strategies with suggestions for addressing power imbalances between Inuit communities and researchers. | Thematic analysis was conducted, which involved familiarization; generating initial codes; and developing, refining and defining themes. | The analysis noted the minimal analysis of the tensions and challenges in assessment of knowledge transfer. |
| Oliver et al. (2014) |  |  | Yes | Yes | Can't tell |
|  | The most frequently reported barriers to evidence uptake were poor access to good quality relevant research, and lack of timely research output. The most frequently reported facilitators were collaboration between researchers and policymakers, and improved relationships and skills. There is an increasing amount of research into new models of knowledge transfer, and evaluations of interventions such as knowledge brokerage. | Not applicable, qualitative themes. | There was a high degree of consistency in the findings, even though studies were from very different contexts. The similarities reported in these studies may be accounted for by the similarity in approach and methods used. | Factors which affected evidence use were coded as barriers or facilitators against a pre-defined list of factors, which was iteratively updated as new factors were identified. | No mention of unintended consequences, negative effects or harms arising from evidence-based decision-making. |

| **Review lead author (year)** | **6. What are the overall results of the review?** | **7. How precise are the results?** | **8. Can the results be applied to the local population?** | **9. Were all important outcomes considered?** | **10. Are the benefits worth the harms and costs?** |
| --- | --- | --- | --- | --- | --- |
| Orton et al. (2011) |  |  | Yes | Yes | Yes |
|  | Decision making processes varied between settings and by key players. There was no reliable evidence on the extent of research use. Its impact was often indirect, competing with other influences. Barriers to research evidence use included: decision makers’ perceptions; the gulf between researchers and decision makers; the culture of decision making; competing influences on decision making; and practical constraints. Suggested (but largely untested) ways of overcoming these included: research targeted at decision makers’ needs; research clearly highlighting key messages; and capacity building. There was little evidence on the role of research evidence in decision making to reduce inequalities. | Not applicable, qualitative themes. | Contradictory findings are explained in terms of study design, methodological quality, and samples and settings accessed. Systematic review limited to countries with universal health care coverage (including: Europe, Canada, Australia and New Zealand). | Data were combined as a narrative review. Data from individual studies were coded and organised according to the main themes identified in the systematic review objectives. | Some consideration of how an evidence-based approach to public health may actually increase health inequalities, as it is likely to reflect the same biases as the production of research evidence. |
| Plamondon et al. (2019) |  |  | Yes | Yes | Yes |
|  | Four distinct kinds of promising practices for connecting KWA for health equity were identified and included: ways of structuring systems, ways of working together, and ways of doing research and ways of doing knowledge translation. Our synthesis reveals that advancing health equity requires greater awareness, dialogue, and action that aligns with the what is known about the causes of health inequities. | Not applicable, qualitative themes. | Findings offer a set of daily tools to support people to engage in a participatory, democratic exercise (connecting knowledge with action) that raises collective awareness about the equity options available in any given situation. | Critical interpretive synthesis (CIS) involves systematic analysis of a nebulous and complex literature using exploratory, rather than hypothetical, research questions. | Some consideration of how using integrated approaches without explicit efforts to mitigate the power implications of class and race would present legitimate risks of doing nothing to advance health equity or, worse, doing harm by masking or perpetuating inequities. |
| Salsberg et al. (2015) |  |  | Can't tell | Yes | Can't tell |
|  | Adapting and applying the “Reliability Tested Guidelines for Assessing Participatory Research Projects” identified five key strategies: developing advisory committees of researchers and intended research users; developing research agreements; using formal and informal group facilitation techniques; hiring co-researchers/partners from community; and ensuring frequent communication. | Not applicable, qualitative themes. | No information provided about the settings/population of the included studies and therefore the applicability of the results. | A deductive qualitative thematic analysis. | No mention of unintended consequences, negative effects or harms arising from participatory research. |
| Wine et al. (2017) |  |  | Yes | Can't tell | Can't tell |
|  | The key components, facilitators, challenges, and best practices for collaborative research were described through seven emerging themes: (a) allocating time and resources, (b) addressing disciplinary and sectoral issues, (c) building relationships, (d) ensuring representation, (e) embedding participation in the research, (f) supporting ongoing collaboration, and (g) developing knowledge translation and exchange. | Not applicable, qualitative themes. | Identified comprehensive details of the key components, facilitators, challenges, and best practices that impact the collaborative research process. | Thematic analysis of the identified data on the collaborative process facilitators, challenges, and best practices. The identified emerging themes are reported. | No mention of unintended consequences, negative effects or harms arising from collaborative research. |

**References**

ARMSTRONG, R., WATERS, E., DOBBINS, M., ANDERSON, L., MOORE, L., PETTICREW, M., CLARK, R., PETTMAN, T. L., BURNS, C., MOODIE, M., CONNING, R. & SWINBURN, B. 2013. Knowledge translation strategies to improve the use of evidence in public health decision making in local government: intervention design and implementation plan. *Implement Sci,* 8**,** 121.

COHEN, B. E. & MARSHALL, S. G. 2017. Does public health advocacy seek to redress health inequities? A scoping review. *Health Soc Care Community,* 25**,** 309-328.

FARRER, L., MARINETTI, C., CAVACO, Y. K. & COSTONGS, C. 2015. Advocacy for health equity: a synthesis review. *Milbank Q,* 93**,** 392-437.

HAYNES, A., ROWBOTHAM, S. J., REDMAN, S., BRENNAN, S., WILLIAMSON, A. & MOORE, G. 2018. What can we learn from interventions that aim to increase policy-makers' capacity to use research? A realist scoping review. *Health Res Policy Syst,* 16**,** 31.

JAKOBSEN, M. W., EKLUND KARLSSON, L., SKOVGAARD, T. & ARO, A. R. 2019. Organisational factors that facilitate research use in public health policy-making: a scoping review. *Health Res Policy Syst,* 17**,** 90.

KNEALE, D., ROJAS-GARCÍA, A., RAINE, R. & THOMAS, J. 2017. The use of evidence in English local public health decision-making: a systematic scoping review. *Implement Sci,* 12**,** 53.

LORENC, T., TYNER, E. F., PETTICREW, M., DUFFY, S., MARTINEAU, F. P., PHILLIPS, G. & LOCK, K. 2014. Cultures of evidence across policy sectors: systematic review of qualitative evidence. *Eur J Public Health,* 24**,** 1041-7.

MASOOD, S., KOTHARI, A. & REGAN, S. 2020. The use of research in public health policy: a systematic review. *Evidence & Policy: A Journal of Research, Debate and Practice,* 16**,** 7-43.

MCDONALD, M. E., PAPADOPOULOS, A., EDGE, V. L., FORD, J., SUMNER, A. & HARPER, S. L. 2016. What do we know about health-related knowledge translation in the Circumpolar North? Results from a scoping review. *Int J Circumpolar Health,* 75**,** 31223.

OLIVER, K., INNVAR, S., LORENC, T., WOODMAN, J. & THOMAS, J. 2014. A systematic review of barriers to and facilitators of the use of evidence by policymakers. *BMC Health Serv Res,* 14**,** 2.

ORTON, L., LLOYD-WILLIAMS, F., TAYLOR-ROBINSON, D., O'FLAHERTY, M. & CAPEWELL, S. 2011. The use of research evidence in public health decision making processes: systematic review. *PLoS One,* 6**,** e21704.

PLAMONDON, K. M., CAXAJ, C. S., GRAHAM, I. D. & BOTTORFF, J. L. 2019. Connecting knowledge with action for health equity: a critical interpretive synthesis of promising practices. *Int J Equity Health,* 18**,** 202.

SALSBERG, J., PARRY, D., PLUYE, P., MACRIDIS, S., HERBERT, C. P. & MACAULAY, A. C. 2015. Successful strategies to engage research partners for translating evidence into action in community health: a critical review. *J Environ Public Health,* 2015**,** 191856.

WINE, O., AMBROSE, S., CAMPBELL, S., VILLENEUVE, P. J., BURNS, K. K. & OSORNIO-VARGAS, Á. R. 2017. Key Components of Collaborative Research in the Context of Environmental Health: A Scoping Review. *Journal of Research Practice,* 13**,** 2.
