# Supplementary material for: Strategies for knowledge exchange for action to address place-based determinants of health inequalities: an umbrella review
Source: J Public Health (Oxf). 2022 Nov 30;45(3):e467–77. doi: 10.1093/pubmed/fdac146 (PMC10470361; doi:10.1093/pubmed/fdac146)
Supplement: Supplementary_file_6_-_Barriers_and_Facilitators_to_KE_fdac146 [file supplementary_file_6_-_barriers_and_facilitators_to_ke_fdac146.docx]

**Supplementary file: The barriers and facilitators to knowledge exchange reported by the included studies**

*(Please see the end of the file for sample size calculations)*

**Part A: Reported barriers and facilitators for exchanging research evidence and data**

**Key: Research; Data**

| **Characteristic** | **Source** | **Collaborative Models** | **Participatory models** | **KE as part of advocacy** | **Evidence access** |
| --- | --- | --- | --- | --- | --- |
| **Individuals – Barriers** | *Review level* | *Lack of policymaker research skills:* LORENC; LORENC  *Lack of community member recruitment/ engagement*:  WINE | *Lack of community member recruitment/ engagement*:  WINE |  | *Lack of policymaker research skills:* LORENC; LORENC |
|  | *Study level* | *Lack of researcher KE skills:*  HAY-OLI 01  *Lack of policymaker research skills:* HAY-OLI 01 |  |  | *Lack of researcher KE skills:*  HAY-OLI 01; KNE-05  *Lack of policymaker research skills:*  HAY-OLI 01 |
| **Individuals – Facilitators** | *Review level* | *Researcher professional standing:*  LORENC; LORENC  *Policymaker research skills:*  LORENC; LORENC |  |  | *Researcher professional standing:*  LORENC; LORENC  *Policymaker research skills:*  LORENC; LORENC |
|  | *Study level* | *Policymaker research skills:* ORT-01 | *Community member recruitment/ engagement*: FAR-02 |  | *Policymaker research skills:* ORT-01  *Community member recruitment/ engagement:* FAR-02 |
| **Knowledge-**  **Barriers** | *Review level* | *Volume of research:* LORENC; LORENC  *Lack of relevance, timeliness:* LORENC; LORENC  *Low quality, credibility:* LORENC; LORENC  *Format/ presentation of findings:* LORENC; LORENC  Ownership of findings: *WINE* | Ownership of findings: *WINE* |  | *Volume of research:* LORENC; LORENC  *Lack of relevance, timeliness:* LORENC; LORENC  *Low quality, credibility:* LORENC; LORENC  *Format/ presentation of findings:*  LORENC; LORENC |
|  | *Study level* | *Lack of relevance, timeliness:*  HAY-OLI- 01, KNE-03; OLI-WIN-01  *Low quality, credibility:* OLI-WIN-01  *Lack of Infrastructure to access findings:* OLI-WIN-01  *Evidence hierarchy:* OLI-WIN-01 | *Lack of relevance, timeliness:*  OLI-WIN-01  *Low quality, credibility:* OLI-WIN-01  *Lack of infrastructure to access findings:* OLI-WIN-01  *Evidence hierarchy:* OLI-WIN-01 | *Evidence hierarchy:* COH-01,  COH-01 | *Relevance, timeliness:* HAY-OLI- 01; KNE-04; KNE-04; KNE-05; OLI-ORT-01  *Quality, credibility:* OLI-ORT-01;  *Evidence hierarchy:* KNE-04; KNE-04; OLI-ORT-01 |
| **Knowledge Facilitators** | *Review level* | *Quality, credibility:* LORENC; LORENC  *Format/ presentation of findings:*  LORENC; LORENC  *Co-produced research design: WINE*  *Relevance, timeliness: WINE*  Ownership of findings: *WINE Dissemination plan: WINE*  *Format/ presentation of findings: WINE* | *Co-produced research design: WINE*  *Relevance, timeliness: WINE*  Ownership of findings: *WINE*  *Dissemination plan: WINE*  *Format/ presentation of findings: WINE* |  | *Quality, credibility:*  LORENC; LORENC  *Format/ presentation of findings*  LORENC; LORENC |
|  | *Study level* | *Relevance, timeliness:*  HAY-OLI- 01; KNE-01; MAS-02  *Quality, credibility:*  HAY-OLI- 01; MAS-02  *Dissemination:* MAS-02 |  |  | *Relevance, timeliness:*  FAR-OLI-01; HAY-OLI- 01; KNE-05; MAS-02; OLI-02; OLI-05  *Quality, credibility:*  FAR-OLI-01; HAY-OLI- 01; MAS-02; OLI-05  *Infrastructure to access findings:*  FAR-OLI-01; KNE-05; OLI-02  *Format/ presentation of findings:*  FAR-OLI-01; KNE-05; OLI-05  *Dissemination:* FAR-OLI-01; MAS-02 |
| **Relationships- Barriers** | *Review level* | *Power imbalance:* WINE  *Lack of intersectoral working/thinking:* WINE  *Lack of 2 communication, collaboration:* WINE  *Previous bad experience:* WINE | *Power imbalance:* WINE  *Lack of intersectoral working/thinking:* WINE  *Lack of 2 communication, collaboration:* WINE  *Previous bad experience:* WINE |  |  |
|  | *Study level* | *Lack of intersectoral working/thinking:* HAY-OLI 01 |  | *Lack of intersectoral working/thinking:*  FAR-PLA-01 | *Lack of intersectoral working/thinking:*  FAR-PLA-01; HAY-OLI 01 |
| **Relationships- Facilitators** | *Review level* | *Power balance:* WINE  *Intersectoral working/thinking: W*INE  Two-way communication/ collaboration: WINE  *Trust:* WINE  *Roles undertaking KE work:*  WINE (knowledge brokers)  *Leadership:* WINE | *Power balance:* WINE  *Intersectoral working/thinking:* WINE  Two-way communication/ collaboration: WINE  *Trust:* WINE  *Roles undertaking KE work:* WINE (knowledge brokers)  *Leadership*: WINE |  |  |
|  | *Study level* | *Intersectoral working/thinking:*  KNE-03  *Two communication, collaboration:*  HAY-OLI 01; KNE-03;  *Trust:* KNE-03  *Long term:* KNE-03 | *Tailoring to community context:* McD-03  *Roles undertaking KE work:*  COH-04 (community health workers)  COH-04 (community health workers)  *Networks*: FAR-02 | *Roles undertaking KE:*  COH-04 (community health workers)  COH-04 (community health workers) | *Tailoring to community context:* McD-03  *Networks:* FAR-02  *Two communication, collaboration:*  HAY-OLI-01  *Long term:*  OLI-ORT-01; FAR-01  *Contacts, connections:*  OLI-ORT-01 |
| **Resources - Barriers** | *Review level* | *Lack of time, opportunity:*  LORENC; LORENC; WINE  *Lack of training:* WINE | *Lack of time, opportunity:* WINE  *Lack of training:* WINE |  | *Lack of time, opportunity:*  LORENC; LORENC |
|  | *Study level* | *Lack of finances:* MAS-02, ORT-01  *Lack of time, opportunity:* ORT-01 |  |  | *Lack of finances:* MAS-02; OLI-ORT-01; ORT-01  *Lack of time, opportunity*: OLI-ORT-01; ORT-01 |
| **Resources - Facilitators** | *Review level* | *Timing/ opportunity:* WINE  *Financial cost:* WINE  *Training:* WINE | *Timing/ opportunity:* WINE  *Financial cost:* WINE  *Training:* WINE |  |  |
|  | *Study level* | *Financial:* KNE-03  *Time, opportunity:* KNE-03 |  |  |  |
| **Organisational & macro - Barriers** | *Review level* | *Lack of organisational support:*  LORENC; LORENC |  |  | *Lack of organisational support:*  LORENC; LORENC |
|  | *Study level* | *Competing interests:* ORT-01  Culture, inertia: KNE-01  *Political context:* KNE-01, ORT-01 |  | *Competing interests:*  FAR-PLA-01  *Political context:* FAR-PLA-01 | *Competing interests:* FAR-PLA-01; OLI-ORT-01; ORT-01  *Short-term flux:* KNE-05  *Political context:* FAR-PLA-01; KNE-04; KNE-04; KNE-05; OLI-ORT-01 |
| **Organisational & macro -**  **Facilitators** | *Review level* | *Political context:* LORENC; LORENC; WINE  *Senior support:* WINE  *Decision making/policy processes:* WINE  *Long term planning / support*: WINE *Rewards & recognition*: WINE | *Senior support:* WINE  *Decision making/policy processes:* WINE  *Long term planning / support*: WINE *Rewards & recognition*: WINE |  | *Political context:*  LORENC; LORENC |
|  | *Study level* | *Alignment with organisational goals:* KNE-01  *Legal support:* OLI-WIN-01 | *Legal support:* OLI-WIN-01 | Policy windows: FAR-PLA-01 | *Short-term flux:* KNE-05  *Political context:* KNE-04; KNE-04; OLI-ORT-01; ORT-01  *Policy windows:* FAR-PLA-01 |

**Part B: Reported barriers and facilitators for exchanging lay or policymaker/practitioner forms of knowledge**

**Key:** **Lay knowledge; policymaker/practitioner knowledge**

| **Characteristic** | **Source** | **Collaborative Models** | **Participatory models** | **KE as part of advocacy** | **Evidence access** |
| --- | --- | --- | --- | --- | --- |
| **Individuals – Barriers** | *Review level* | *Lack of policymaker research skills:* LORENC  *Lack of community member recruitment/ engagement*:  WINE; WINE | *Lack of community member recruitment/ engagement*:  WINE; WINE |  | *Lack of policymaker research skills:* LORENC |
|  | *Study level* |  |  | *Researcher attitudes towards advocacy:* COH-PLA-01 |  |
| **Individuals – Facilitators** | *Review level* | *Researcher professional standing:*  LORENC  *Policymaker research skills:* LORENC |  |  | *Researcher professional standing:*  LORENC  *Policymaker research skills:* LORENC |
|  | *Study level* |  | *Community member recruitment/ engagement:* FAR-02; McD-02 |  | *Community member recruitment/ engagement:* FAR-02; McD-02 |
| **Knowledge- Barriers** | *Review level* | *Volume of research:* LORENC  *Lack of relevance, timeliness:* LORENC  *Low quality, credibility:* LORENC  *Format/ presentation of findings:*  LORENC  *Ownership of findings*: WINE; WINE | *Ownership of findings*: *WINE; WINE* |  | *Volume of research:* LORENC  *Lack of relevance, timeliness:*  LORENC  *Low quality, credibility:* LORENC  *Format/ presentation of findings:*  LORENC |
|  | *Study level* | *Relevance, timeliness:* OLI-WIN-01  *Quality, credibility:* OLI-WIN-01  *Infrastructure to access findings:*  OLI-WIN-01  *Evidence hierarchy:* OLI-WIN-01 | *Relevance, timeliness*: OLI-WIN-01  *Quality, credibility:* OLI-WIN-01  *Infrastructure to access findings:*  OLI-WIN-01  *Evidence hierarchy*: OLI-WIN-01 |  | *Relevance, timeliness*: KNE-04  *Evidence hierarchy:* KNE-04 |
| **Knowledge – Facilitators** | *Review level* | *Quality, credibility:* LORENC  *Format/ presentation of findings:*  LORENC; *WINE; WINE*  *Co-produced research design: WINE; WINE*  *Co-produced research design: WINE; WINE*  *Relevance, timeliness: WINE; WINE*  Ownership of findings: *WINE; WINE*  *Dissemination plan: WINE; WINE* | *Co-produced research design: WINE; WINE*  *Relevance, timeliness: WINE; WINE*  Ownership of findings: *WINE; WINE*  *Dissemination plan: WINE; WINE*  *Format/ presentation of findings: WINE; WINE* |  | *Quality, credibility:* LORENC  *Format/ presentation of findings:*  LORENC |
|  | *Study level* |  |  |  |  |

| **Relationships- Barriers** | *Review level* | *Power balance:* WINE; WINE  *Interdisciplinary working/ thinking:* WINE; WINE  *Lack of 2 communication, collaboration:* WINE; WINE  Previous poor experience: WINE; WINE | *Power balance:* WINE; WINE  *Interdisciplinary working/ thinking:* WINE; WINE  *Lack of 2 communication, collaboration:* WINE; WINE  Previous poor experience: WINE; WINE |  |  |
| --- | --- | --- | --- | --- | --- |
|  | *Study level* | *Intersectoral working/thinking:* COH-05; COH-05  *Lack of two communication, collaboration:* COH-05; COH-05 |  | *Intersectoral working/thinking:* COH-05; COH-05  *Lack of two communication, collaboration:* COH-05; COH-05 |  |
| **Relationships- Facilitators** | *Review level* | *Power balance:* WINE; WINE  *Intersectoral working/thinking: W*INE; WINE  Two-way communication/ collaboration: WINE; WINE  *Trust:* WINE; WINE  *Roles undertaking KE work:* WINE (knowledge brokers); WINE (knowledge brokers);  Leadership: WINE; WINE | *Power balance:* WINE; WINE  *Intersectoral working/thinking: W*INE; WINE  Two-way communication/ collaboration: WINE; WINE  *Trust:* WINE; WINE  *Roles undertaking KE work:* WINE (knowledge brokers); WINE (knowledge brokers);  Leadership: WINE; WINE |  |  |
|  | *Study level* |  | *Tailoring to community context:*  McD-02; McD-03  *Roles undertaking KE work:*  COH-04 (community health workers)  *Networks:* FAR-02  *Trust:* McD-02 | *Roles undertaking KE work:*  COH-04 (community health workers)  *Long term:* FAR-04 | *Tailoring to community context:*  McD-02; McD-03  *Networks:* FAR-02  *Trust:* McD-02  *Long term relationships:* FAR-01 |
| **Resources**  **- Barriers** | *Review level* | *Lack of time, opportunity:*  LORENC; WINE; WINE  *Lack of Training*: WINE; WINE | *Lack of time, opportunity:* WINE; WINE  *Lack of training:* WINE; WINE |  | *Lack of time, opportunity:* LORENC |
|  | *Study level* |  |  |  |  |
| **Resources - Facilitators** | *Review level* | *Timing/ opportunity*: WINE; WINE *Financial cost:* WINE; WINE  *Training:* WINE; WINE | *Timing/ opportunity:* WINE; WINE *Financial cost:* WINE; WINE  *Training:* WINE; WINE |  |  |
|  | *Study level* |  |  |  |  |
| **Organisational & macro - Barriers** | *Review level* | *Lack of organisational support:*  LORENC |  |  | *Lack of organisational support:*  LORENC |
|  | *Study level* |  |  |  | *Political context:* KNE-04 |

| **Organisational**  **& macro -**  **Facilitators** | *Review level* | *Political context:* LORENC  *Senior support:* WINE; WINE  *Decision making/policy processes:* WINE; WINE  *Long term planning/ support*: WINE; WINE  *Rewards & recognition*: WINE; WINE | *Senior support:* WINE; WINE  *Decision making/policy processes:* WINE; WINE  *Long term planning/ support*: WINE; WINE  *Rewards & recognition*: WINE; WINE |  | *Political context:* LORENC |
| --- | --- | --- | --- | --- | --- |
|  | *Study level* | *Legal support:* OLI-WIN-01 | *Legal support:* OLI*-*WIN-01 | *Policy windows:* FAR-04 | *Political context:* KNE-04 |

**Part C: Reported barriers and facilitators for exchanging other forms of knowledge**

| **Characteristic** | **Source** | **Collaborative Models** | **Participatory models** | **KE as part of advocacy** | **Evidence access** |
| --- | --- | --- | --- | --- | --- |
| **Individuals – Barriers** | *Review level* | *Lack of policymaker research skills:* LORENC  *Lack of community member recruitment/ engagement*:  WINE | *Lack of community member recruitment/ engagement*:  WINE |  | *Lack of policymaker research skills:* LORENC |
|  | *Study level* |  |  | *Researcher attitudes towards advocacy:* COH-PLA-01; FAR-03 |  |
| **Individuals – Facilitators** | *Review level* | *Researcher professional standing:*  LORENC  *Policymaker research skills:* LORENC |  |  | *Researcher professional standing:*  LORENC  *Policymaker research skills:* LORENC |
|  | *Study level* |  |  |  |  |
| **Knowledge**  **- Barriers** | *Review level* | *Volume of research:* LORENC  *Lack of relevance, timeliness:* LORENC  *Low quality, credibility:* LORENC  *Format/ presentation of findings:*  LORENC  Ownership of findings: *WINE* | *Ownership of findings*: WINE |  | *Volume of research:* LORENC  *Lack of relevance, timeliness:*  LORENC  *Low quality, credibility:* LORENC  *Format/ presentation of findings*  LORENC |
|  | *Study level* | *Relevance, timeliness:* KNE-03; OLI-WIN-01  *Quality, credibility:* OLI-WIN-01  *Infrastructure to access findings:*  OLI-WIN-01  *Evidence hierarchy:* OLI-WIN-01 | *Relevance, timeliness:* OLI-WIN-01  *Quality, credibility:* OLI-WIN-01  *Infrastructure to access findings:*  OLI-WIN-01  *Evidence hierarchy:* OLI-WIN-01 |  | *Relevance, timeliness:* OLI-03  *Quality, credibility:* OLI-03  *Infrastructure to access findings:*  OLI-03  *Evidence hierarchy:* OLI-03 |
| **Knowledge - Facilitators** | *Review level* | *Quality, credibility:* LORENC  *Format/ presentation of findings:*  LORENC  *Co-produced research design: WINE*  *Relevance, timeliness: WINE*  Ownership of findings: *WINE*  *Dissemination plan: WINE*  *Format/ presentation of findings: WINE* | *Co-produced research design: WINE*  *Relevance, timeliness: WINE*  Ownership of findings: *WINE*  *Dissemination plan: WINE*  *Format/ presentation of findings: WINE* |  | *Quality, credibility:* LORENC  *Format/ presentation of findings:*  LORENC |
|  | *Study level* | *Relevance, timeliness:* MAS-02  *Quality, credibility:* MAS-02  *Dissemination:* MAS-02 |  |  | *Relevance, timeliness:* MAS-02; OLI-02  *Quality, credibility:* MAS-02  *Dissemination:* MAS-02; OLI-02 |

| **Relationships- Barriers** | *Review level* | *Power imbalance:* WINE  *Lack of intersectoral working/thinking:* WINE  *Lack of 2 communication, collaboration:* WINE  *Previous bad experience:* WINE | *Power imbalance:* WINE  *Lack of intersectoral working/thinking:* WINE  *Lack of 2 communication, collaboration:* WINE  *Previous bad experience:* WINE |  |  |
| --- | --- | --- | --- | --- | --- |
|  | *Study level* |  |  | *Intersectoral working/thinking:*  FAR-PLA-01 | *Intersectoral working/thinking:*  FAR-PLA-01 |
| **Relationships- Facilitators** | *Review level* | *Power balance:* WINE  *Intersectoral working/thinking: W*INE  Two-way communication/ collaboration: WINE  *Trust:* WINE  *Roles undertaking KE work:*  WINE (knowledge brokers)  *Leadership:* WINE | *Power balance:* WINE  *Intersectoral working/thinking:* WINE  Two-way communication/ collaboration: WINE  *Trust:* WINE  *Roles undertaking KE work:* WINE (knowledge brokers)  *Leadership*: WINE |  |  |
|  | *Study level* |  | *Roles undertaking KE work:*  COH04 (community health workers) | *Roles undertaking KE work:*  COH04 (community health workers)  FAR-03 (policy champions)  *Long term:* KNE-04 |  |
| **Resources**  **- Barriers** | *Review level* | *Lack of time, opportunity:*  LORENC; WINE | *Lack of time, opportunity:* WINE  *Lack of training:* WINE |  | *Lack of time, opportunity:*  LORENC |
|  | *Study level* | *Lack of finances:* MAS-02 |  |  | *Lack of finances:* MAS-02 |
| **Resources - Facilitators** | *Review level* | *Timing/ opportunity:* WINE  *Financial cost:* WINE  *Training:* WINE | *Timing/ opportunity:* WINE  *Financial cost:* WINE  *Training:* WINE |  |  |
|  | *Study level* |  |  |  |  |
| **Organisational & Macro - Barriers** | *Review level* | *Lack of organisational support:*  LORENC |  |  | *Lack of organisational support:*  LORENC |
|  | *Study level* |  |  | *Competing interests:* FAR-PLA-01  *Political context:* FAR-03 | *Competing interests:* FAR-PLA-01 |
| **Organisational & Macro -**  **Facilitators** | *Review level* | *Political context:* LORENC  *Senior support:* WINE  *Decision making/policy processes:* WINE  *Long term planning / support*: WINE *Rewards & recognition*: WINE | *Senior support:* WINE  *Decision making/policy processes:* WINE  *Long term planning / support*: WINE *Rewards & recognition*: WINE |  | *Political context:*  LORENC |
|  | *Study level* | *Legal support:* OLI-WIN-01 | *Legal support:* OLI-WIN-01 | *Policy windows:* FAR-03; FAR-PLA-01KNE-04 | *Policy windows:* FAR-PLA-01 |

**Part E: Reported barriers and facilitators for exchanging knowledge (model of knowledge exchange or knowledge type unclear)**

**Key: Research; Data; Community; Other**

| **Characteristic** | **Source** | **Unspecified models** | **Active KE (knowledge type unclear)** |
| --- | --- | --- | --- |
| **Individuals – Barriers** | *Study level* | Policy maker r*esearch training/ skills:* MAS-01; MAS-01; MAS-01; |  |
| **Individuals – Facilitators** | *Study level* | *Researcher KE training/ skills:*  FAR-KNE-01  Policymaker r*esearch training/ skills:*  FAR-KNE-01; OLI-04; OLI-04  *Policymaker evidence based policy making:* MAS-01; MAS-01; MAS-01; |  |
| **Knowledge - Barriers** | *Study level* | *Relevance, timeliness:* FAR-KNE-01; KNE-02; KNE-02; KNE-02; OLI-01  *Infrastructure to access findings:* MAS-01; MAS-01; MAS-01; OLI-01  *Format/ presentation of findings:* OLI-01  *Evidence hierarchy:* FAR-KNE-01 |  |
| **Knowledge - Facilitators** | *Study level* | *Relevance, timeliness:* FAR-KNE-01;  KNE-02; KNE-02; KNE-02 KNE-OLI-01; KNE-OLI-01  *Infrastructure to access findings:* MAS-01; MAS-01; MAS-01; JAK-01  *Evidence hierarchy:* MAS-01; MAS-01; MAS-01; OLI-04; OLI-04  *Dissemination:* OLI-04; OLI-04 |  |
| **Relationships – Barriers** | *Study level* | *Contact, connections:* FAR-KNE-01 |  |
| **Relationships – Facilitators** | *Study level* | *Intersectoral working/thinking:*  FAR-KNE-01  *Contact, connections:* OLI-04; OLI-04 JAK-01 | *2-way communication/ collaboration:* COH-06 |
| **Resources – Barriers** | *Study level* |  |  |
| **Resources – Facilitators** | *Study level* | *Personnel available:* OLI-04; OLI-04  *Decision making/policy processes*  JAK-01 |  |
| **Organisational & Macro - Barriers** | *Study level* | *Culture:* MAS-01; MAS-01; MAS-01  *Decision making processes:* FAR-KNE-01 |  |
| **Organisational**  **& Macro -**  **Facilitators** | *Study level* | *Culture:* MAS-01; MAS-01; MAS-01  *Absence of competing interests:*  KNE-OLI-01; KNE-OLI-01 |  |

**Part F: Number of sources from which barriers and facilitators information was extracted by knowledge exchange approach**

| **Sources** | | **Collaborative Models** | **Participatory models** | **KE as part of advocacy** | **Evidence access** | **Unclear** |
| --- | --- | --- | --- | --- | --- | --- |
| Studies | Full focus | KNE-01  KNE-03 | - | COH-03  COH-PLA-01  FAR-03  FAR-04 | FAR-01  FAR-OLI-01  KNE-04  KNE-05  OLI-02  OLI-03  OLI-05  OLI-ORT-01 | FAR-KNE-01  JAK-01  OLI-01  OLI-04  KNE-OLI-01  KNE-02  MAS-01 |
|  | Full - total | 2 | 0 | 4 | 8 | 7 |
|  | Partial focus | COH-05  HAY-OLI-01  MAS-02  OLI-WIN-01  ORT-01 | COH-04  FAR-02  McD-02  McD-03  OLI-WIN-01 | COH-04  COH-05  FAR-PLA-01 | FAR-02  FAR-PLA-01  HAY-OLI-01  MAS-02  McD-02  McD-03  ORT-01 |  |
|  | Partial - total | 5 | 5 | 3 | 7 | 0 |
| Reviews | Full focus |  |  |  |  |  |
|  | Partial focus | LORENC (2014)  WINE (2017) | WINE (2017) | - | LORENC (2014) |  |
|  | Partial - total | 2 | 1 | 0 | 1 | 0 |
| Total |  | 9 | 6 | 7 | 15 | 7 |

***Source where approach is unclear*** COH-06: ‘Active knowledge exchange’
