# Supplementary material for: Strategies for knowledge exchange for action to address place-based determinants of health inequalities: an umbrella review
Source: J Public Health (Oxf). 2022 Nov 30;45(3):e467–77. doi: 10.1093/pubmed/fdac146 (PMC10470361; doi:10.1093/pubmed/fdac146)
Supplement: Supplementary_file_3b_-_Included_Study_Characteristics_fdac146 [file supplementary_file_3b_-_included_study_characteristics_fdac146.docx]

**Supplementary file 3b: Included Study Characteristics**

| **Study reference** | **Systematic review lead author (date)** | **Primary study lead author (date)** | **Study aim/research question** | **Study design** | **Country/ies and region** | **Setting** | **Place based determinant(s)** | **Participants (sample size)** | **Quality appraisal (as assessed by authors)** |
| --- | --- | --- | --- | --- | --- | --- | --- | --- | --- |
| ARM-01 | Armstrong (2013) | Amara (2004) ^(1)^ | Not reported in review | Not reported in review | Not reported in review | Local government | Not reported in review | Not reported in review | Not undertaken |
| COH-01 | Cohen (2017) | Garcia (2015) ^(2)^ | This article provides perspectives from public health professionals who participated in successful public health policy advocacy efforts in their community | Not reported in review | USA | Policy settings | Health equity | Public health professionals (n = not reported) | Not undertaken |
| COH-02 | Cohen (2017) | Giang (2008) ^(3)^ | This article focuses on a key component of a advocacy campaign to address the grocery gap in underserved communities: the creation of an evidence-based report that served as a strong, credible foundation for the campaign | Case study | USA | Low income neighbourhoods | Access to food | Underserved communities (n = not reported) | Not undertaken |
| COH-03 | Cohen (2017) | Gomm (2006) ^(4)^ | This paper describes a public health advocacy framework. It then presents a case study of its application for planning community action by a community alliance concerned about increased heavy traffic on roads. | Case study | Australia | Community | Transportation | Members of a community alliance (n = not reported) | Not undertaken |
| COH-04 | Cohen (2017) | Ingram (2014) ^(5)^ | This paper describes preliminary results of Acción para la Salud, a public health intervention in which Community health workers (CHWs) from five health agencies engaged their community in the process of making positive systems and environmental changes. | Not reported in review | USA | Community | Social determinants of health in general | Community health workers from 5 health agencies; community members; researchers (n = not reported) | Not undertaken |
| COH-05 | Cohen (2017) | McAndrews (2014) ^(6)^ | To study one community that advocated to improve community health through the design and reconstruction of an arterial road in their neighborhood. | Focus groups, interviews and participatory photo-mapping | Australia | Community | Transportation | Residents of neighbourhood (n = not reported) | Not undertaken |
| COH-06 | Cohen (2017) | Tsoukalas (2003) ^(7)^ | To investigate the tobacco industry’s strategies to fight local tobacco control efforts in Duluth, Minn. | Case study | USA | Urban location | Tobacco control | Not reported in review | Not undertaken |
| COH-PLA-01 | Cohen (2017), Plamondon (2019) | Brassolotto (2014) ^(8)^ | To understand how differences in addressing the social determinants of health (SDH) among health units | Interviews | Canada | Public health units | Social determinants of health in general | Medical Officers of Health and lead staff persons (n = not reported) | Quality score 6 (10 is highest) applied by review authors |
| FAR-01 | Farrar (2015 | Roos (2010) ^(9)^ | Not reported in review | Empirical | Canada | Policy environment | Social determinants of health in general | Researchers, policymakers, school boards, ministry staff; NGOs, private sector, policymakers (n = not reported) | Not undertaken |
| FAR-02 | Farrar (2015) | Izumi (2010) ^(10)^ | Not reported in review | Empirical | USA | Community | Not reported in review | Community–academic partnership, policy makers (n = not reported) | Not undertaken |
| FAR-03 | Farrar (2015) | Macnaughton (2013) ^(11)^ | Not reported in review | Empirical | Canada | Policy environment, advocacy | Housing | Advocacy groups, policy champion, decision makers (n = not reported) | Not undertaken |
| FAR-04 | Farrar (2015) | Nelson (1992) ^(12)^ | Not reported in review | Case study | Canada | Policy environment, advocacy | Housing | Mental health advocates and coalitions, government decision makers (n = not reported) | Not undertaken |
| FAR-KNE-01 | Farrar (2015), Kneale 2017 | Orton (2011) ^(13)^ | Not reported in review | Qualitative | UK (England) | Policy environment | Social determinants of health in general | Policy makers (n = not reported) | Not undertaken |
| FAR-OLI-01 | Farrar (2015), Oliver (2014) | Priest (2009) ^(14)^ | Not reported in review | Case study | International | Policy environment | Social determinants of health in general | Policy advisors, policy makers (n = not reported) | Not undertaken |
| FAR-PLA-01 | Farrar (2015), Plamondon (2019) | Baum (2013) ^(15)^ | Not reported in review | Empirical | Australia | Policy environment | Social determinants of health in general | Health ministers (n = not reported) | Not undertaken |
| HAY-OLI-01 | Haynes (2018), Oliver (2014) | Wehrens (2010) ^(16)^ | Not reported in review | Case study | The Netherlands | Policy environment | Environmental health | Policymakers, managers, and epidemiologists from the city council, regional health services and university staff (n=16) | Not undertaken |
| JAK-01 | Jakobsen (2019) | Fazli (2017) ^(17)^ | 1) To identify the knowledge gaps and other barriers to evidence-based decision-making and policy development related to the built environment 2) To identify the policy development infrastructure, processes and mechanisms needed to drive policy changes in this area. | Case study | Canada | Local government; Other (non-profit organisation and funders) | Built environment (Planning, transportation) | Civil servants, external policy advisors (n = not reported) | Not undertaken |
| KNE-01 | Kneale 2017 | King (2014) ^(18)^ | Investigated the use of Social Return on Investment (SROI) evidence in LA decision-making around investing in services to increase levels of physical activity. | Survey and interviews | UK (England) | Local government | Leisure and recreation | Not reported in review | Not undertaken |
| KNE-02 | Kneale 2017 | McGill (2015) ^(19)^ | The study explores how information and evidence are defined, assessed and utilised by local professionals situated beyond the health sector, but whose decisions potentially affect health | Focus groups | Multiple (England, Brazil, USA and Canada) | Local government | Built environment | Local professionals situated beyond the health sector, but whose decisions potentially affect health (n = not reported) | Not undertaken |
| KNE-03 | Kneale 2017 | Milton (2014) ^(20)^ | Taking housing as a case study, the authors aimed to identify the factors contributing to successful evaluative research in the non-health sector | Interviews | UK | Local government | Housing | Not reported in review | Not undertaken |
| KNE-04 | Kneale 2017 | Phillips (2015) ^(21)^ | To explore how decisions that affect public health are rationalised and enacted through discourses of localism, empiricism and holism. | Ethnographic | UK (England) | Local government | Licensing | Not reported in review | Not undertaken |
| KNE-05 | Kneale 2017 | Willmott (2015) ^(22)^ | This paper describes what Directors of Public Health are making the case for, the components of their case and how they present the case for public health. | Interviews | UK (England) | Local government | Social determinants of health in general | Directors of Public Health (n = not reported) | Not undertaken |
| KNE-OLI-01 | Kneale 2017, Oliver (2014) | Blackman (2012) ^(23)^ | To understand how health inequalities are constructed as an object for policy intervention by considering four framings: politics, audit, evidence and treatment | Interviews | UK (England, Scotland, Wales) | Local government and health services | Social determinants of health in general | Local managers (including directors of Public Health) (n = 197) | Not undertaken |
| LOR-01 | Lorenc (2014) | Allender (2009) ^(24)^ | Town planning and transport professionals’ views about NICE public health guidance | Focus groups | UK | Not reported in review | Bulit environment (Planning; transportation) | Town planning and transport professionals (n = not reported) | Score++ |
| LOR-02 | Lorenc (2014) | Allison (2011) ^(25)^ | Barriers and facilitators of community youth violence prevention programmes | Interviews | USA | Not reported in review | Community safety | Not reported in review | Score- |
| LOR-03 | Lorenc (2014) | Coote (2004) ^(26)^ | Role of evidence and evaluation in social policy; policy-makers’ and practitioners’ views and experiences of evaluation (particularly New Deal for Communities) | Case studies and interviews | Mulitple (UK and USA) | Urban location | Urban regeneration and social policy | Policy makers and practitioners (n = not reported) | Score+ |
| LOR-04 | Lorenc (2014) | Freeman (2011) ^(27)^ | Policy development in healthy built environments; research-policy partnerships | Interviews | Australia | Not reported in review | Built environment | Not reported in review | Score+ |
| LOR-06 | Lorenc (2014) | Gudmundsson (2012) ^(28)^ | Use of decision support in transport planning and policy | Case studies and interviews | Sweden | Not reported in review | Transportation | Not reported in review | Score- |
| LOR-07 | Lorenc (2014) | Harrison (2002) ^(29)^ | Practitioners’ views and practices about nature conservation in brownfield sites | Interviews | UK (England) | Not reported in review | Planning; nature conservation | Practitioners (n = not reported) | Score- |
| LOR-08 | Lorenc (2014) | Hatzopoulou (2009) ^(30)^ | Use of modelling tools in decision-making | Interviews | Canada | Policy environment | Transportation | Not reported in review | Score+ |
| LOR-09 | Lorenc (2014) | Hewson (2010) ^(31)^ | Description of course on evidence-based practice for practitioners | Survey | UK | Not reported in review | Transportation (road injury reduction) | Practitioners (n = not reported) | Score- |
| LOR-10 | Lorenc (2014) | Hinchcliff (2010) ^(32)^ | Policy-makers’ views of evidence; evidence–policy relation more generally | Interviews | Mulitple (Australia and USA) | Not reported in review | Transportation | Policy makers (n = not reported) | Score++ |
| LOR-11 | Lorenc (2014) | Marsden (2011) ^(33)^ | Policy transfer in sustainable urban transport | Interviews | Multiple (France, UK, Germany, Sweden, Denmark, USA, Canada) | Not reported in review | Transportation | Not reported in review | Score- |
| LOR-12 | Lorenc (2014) | Minkler (2010) ^(34)^ | Process evaluation of community-based environmental justice advocacy project | Case studies and interviews | USA | Community | Environmental health; planning | Communities (n = not reported) | Score+ |
| LOR-13 | Lorenc (2014) | Petersen (2006) ^(35)^ | Process evaluation of community-based environmental justice advocacy project | Case studies and interviews | USA | Community | Environmental health; planning | Communities (n = not reported) | Score+ |
| LOR-14 | Lorenc (2014) | Sandstrom (2006) ^(36)^ | Urban planners’ views about green space | Interviews | Sweden | Not reported in review | Built environment (Planning) | Urban planners (n = not reported) | Score- |
| LOR-15 | Lorenc (2014) | Timms (2011) ^(37)^ | Policy transfer and policy-makers’ information needs | Interviews | Multiple (France, UK, Italy, Poland, Sweden, Austria, Norway) | Not reported in review | Transportation | Policy makers (n = not reported) | Score- |
| MAS-01 | Masood (2020) | Armstrong (2014) ^(38)^ | To identify the types of evidence and how each contributes to evidence informed decision making process that takes place within local governments | Survey and interviews | Australia | Local government | Social determinants of health in general | Professionals involved in public health planning, policy or programmes (n =135) | Review authors report study met all methodological criteria for mixed method studies (p.16) |
| MAS-02 | Masood (2020) | Francis (2015) ^(39)^ | To examine the needs of decision makers that are linked to promoting the use of evidence synthesis in their practice, and to discuss the implications of this for reviews and the steps that can be taken to build capacity | Interviews | UK | Policy environment | Social determinants of health in general | Public health decision makers in policy organisations (15) and review authors (13) | Review authors not that study did not provide sufficient details about the methods to properly assess its methodological quality (p.16) |
| McD-01 | McDonald 2017 | Jardine (2004) ^(40)^ | Not reported in review | Case study | Canada | Community | Environmental health | Not reported in review | Not undertaken |
| McD-02 | McDonald 2017 | Pufall (2012) ^(41)^ | To determine how residents of the Inuit community of Nain, Nunatsiavut, Canada would like research results disseminated to their community | Focus groups and interviews | Canada | Community | Environmental health | Hunters and general community members (n = not reported) | Not undertaken |
| McD-03 | McDonald 2017 | Tyrrell (2006) ^(42)^ | Not reported in review | Case study | Canada | Community | Environmental health | Not reported in review | Not undertaken |
| OLI-01 | Oliver (2014) | Armstrong (2006) ^(43)^ | Not reported in review | Interviews | Not reported in review | Not reported in review | Not reported in review | Not reported in review | Not undertaken |
| OLI-02 | Oliver (2014) | Jennings (2012) ^(44)^ | Not reported in review | Survey | Not reported in review | Policy environment | Transport, environmental health, drugs policy | Policy advisors, policy makers (n = not reported) | Not undertaken |
| OLI-03 | Oliver (2014) | Lavis (2001) ^(45)^ | Not reported in review | Survey and interviews | Canada | Policy environment | Employmnent | Policy makers, NGO workers (n = not reported) | Not undertaken |
| OLI-04 | Oliver (2014) | Smith (2012) ^(46)^ | Not reported in review | Case study | Not reported in review | Policy environment, health services | Health equity | Researchers, physicians, policy advisors, policy makers, other (n = not reported) | Not undertaken |
| OLI-05 | Oliver (2014) | Weitkamp (2012) ^(47)^ | Not reported in review | Focus groups | Not reported in review | Policy environment | Built environment (Planning) | Policy-makers, local authority staff (n = not reported) | Not undertaken |
| OLI-ORT-01 | Oliver (2014), Orton (2011) | Bickford (2008) ^(48)^ | To explore how research is managed, evaluated, and utilised for public health decision making. | Interviews | Canada | Policy environment | Tobacco control | Directors and policy analysts in provincial government, non-government tobacco organisation representatives with a provincial mandate, and individuals working in public health (n=29) | No overall rating provided |
| OLI-WIN-01 | Oliver (2014), Wine (2017) | Jack (2010) ^(49)^ | Not reported in review | Interviews | Canada | Community | Environmental health | Researchers, policy makers, practitioners working within Aboriginal communities (n = not reported) | Not undertaken |
| ORT-01 | Orton (2011) | Green (2000) ^(50)^ | To explore how evidence is utilised and socially constructed in public health strategy planning. | Case study | UK (England) | Policy environment | Transportation (road injury reduction) | Members of Accident Alliances:health authority representatives, local authority staff, health service providers, other statutory services, and voluntary agencies; national “policy leaders (n = not reported) | No overall rating provided |
| ORT-02 | Orton (2011) | Macintyre (2001) ^(51)^ | To examine the quality of evidence underpinning the scientific advisory group to the UK’s Department of Health review of the latest available information on inequalities in health’s emerging recommendations and to identify gaps. | Case study | UK | Policy environment | Health equity | Documents submitted to the Department of Health's review on inequalities | No overall rating provided |
| ORT-03 | Orton (2011) | Taylor-Robinson (2008) ^(52)^ | To explore attitudes to the use of models for coronary heart disease to support decision making for policy and service planning, and to explore issues relating to timescales within which public health decisions are being made. | Interviews | UK (England, Scotland, Wales) | Policy environment, health services | Not reported in review | National, regional and local level decision makers; physicians; academics and voluntary organisations (n=33) | No overall rating provided |
| PLA-01 | Plamondon (2019) | Baum (2010) ^(53)^ | To examine evidence from a rapid appraisal to determine whether a social inclusion initiative is a useful aspect of government action to reduce health inequity. | Case study | Australia | Local government | Social determinants of health in general | Not reported in review | Quality score 5 (10 is highest) applied by review authors |
| PLA-02 | Plamondon (2019) | Cacari-Stone (2014) ^(54)^ | To explore how community-based participatory research (CBPR) responds to two knowledge-to-action challenges: “1) the gap between scientiﬁc evidence and policy action based on evidence, and (2) the difﬁculty of mobilizing civic engagement for policymaking in the United States | Case study | USA | Community | Environmental health | Not reported in review | Quality score 3 (10 is highest) applied by review authors |
| PLA-03 | Plamondon (2019) | Knight (2014) ^(55)^ | To build on the Unnatural Causes campaign by gathering and disseminating recommendations about public health strategies for achieving health equity | Qualitative | USA | Not reported in review | Social determinants of health in general | Public health experts and community leaders (n = not reported) | Quality score 6 (10 is highest) applied by review authors |
| PLA-04 | Plamondon (2019) | Raphael (2014) ^(56)^ | To investigate how individual characteristics of Medical Officers of Health (MOH) and unit staff (e.g. background training, personal experiences and understandings of the SDH), community features (e.g. urban versus rural, political climate and governance structures) and organizational features (e.g. central versus devolved SDH structures, leadership and training) account for these differences in PHUs’ SDH activities | Qualitative | Canada | Public health units | Social determinants of health in general | Public health leaders and workers (n = not reported) | Quality score 6 (10 is highest) applied by review authors |
| PLA-05 | Plamondon (2019) | Raphael (2015) ^(57)^ | To consider the factors that shape local PHU action on the SDH through a critical realist analysis | Interviews | Canada | Public health units | Social determinants of health in general | Public health leaders and workers (n = not reported) | Quality score 10 (10 is highest) applied by review authors |
| SAL-01 | Salsberg (2015) | Israel (2006) ^(58)^ | Not reported in review | Not reported in review | Not reported in review | Community | Environmental health | Urban residents (n = not reported) | Not undertaken |
| SAL-02 | Salsberg (2015) | Israel (2001) ^(59)^ | Not reported in review | Not reported in review | USA | Community | Not reported in review | Not reported in review | Not undertaken |
| SAL-03 | Salsberg (2015) | Schulz (2001) ^(60)^ | Not reported in review | Not reported in review | Not reported in review | Not reported in review | Not reported in review | Not reported in review | Not undertaken |
| SAL-04 | Salsberg (2015) | Schulz (2002) ^(61)^ | Not reported in review | Not reported in review | Not reported in review | Community | Social determinants of health in general | Not reported in review | Not undertaken |
| SAL-WIN-01 | Salsberg (2015), Wine (2017) | Israel (2005) ^(62)^ | Not reported in review | Not reported in review | Not reported in review | Community | Environmental health | Not reported in review | Not undertaken |
| SAL-WIN-02 | Salsberg (2015), Wine (2017) | Minkler (2006) ^(63)^ | Not reported in review | Case study | USA | Community | Environmental health | Not reported in review | Not undertaken |
| SAL-WIN-03 | Salsberg (2015), Wine (2017) | Minkler (2008) ^(64)^ | Not reported in review | Not reported in review | Not reported in review | Community | Environmental health | Not reported in review | Not undertaken |
| WIN-01 | Wine (2017) | Angelstam (2013) ^(65)^ | Not reported in review | Not reported in review | Sweden | Not reported in review | Environmental health | Not reported in review | Not undertaken |
| WIN-02 | Wine (2017) | Arcury (2001) ^(66)^ | Not reported in review | Not reported in review | USA | Community | Environmental health | Not reported in review | Not undertaken |
| WIN-03 | Wine (2017) | Austin (2010) ^(67)^ | Not reported in review | Not reported in review | USA | Community | Environmental health | Not reported in review | Not undertaken |
| WIN-04 | Wine (2017) | Bharadwaj (2014) ^(68)^ | Not reported in review | Not reported in review | Canada | Community | Environmental health | Not reported in review | Not undertaken |
| WIN-05 | Wine (2017) | Boon (2014) ^(69)^ | Not reported in review | Not reported in review | The Netherlands | Not reported in review | Environmental health | Not reported in review | Not undertaken |
| WIN-06 | Wine (2017) | Brown (2012) ^(70)^ | Not reported in review | Not reported in review | USA | Community | Environmental health | Not reported in review | Not undertaken |
| WIN-07 | Wine (2017) | Burger (2007) ^(71)^ | Not reported in review | Not reported in review | USA | Community | Environmental health | Not reported in review | Not undertaken |
| WIN-08 | Wine (2017) | Burger (2009) ^(72)^ | Not reported in review | Not reported in review | USA | Not reported in review | Environmental health | Not reported in review | Not undertaken |
| WIN-09 | Wine (2017) | Collman (2014) ^(73)^ | Not reported in review | Not reported in review | USA | Community | Environmental health | Not reported in review | Not undertaken |
| WIN-10 | Wine (2017) | Conrad (2013) ^(74)^ | Not reported in review | Not reported in review | USA | Not reported in review | Environmental health | Not reported in review | Not undertaken |
| WIN-11 | Wine (2017) | Corburn (2007) ^(75)^ | Not reported in review | Not reported in review | USA | Not reported in review | Environmental health | Not reported in review | Not undertaken |
| WIN-12 | Wine (2017) | Cummins (2011) ^(76)^ | Not reported in review | Not reported in review | USA | Community | Environmental health | Not reported in review | Not undertaken |
| WIN-13 | Wine (2017) | Downs (2009^(77)^) | Not reported in review | Not reported in review | USA | Community | Environmental health | Not reported in review | Not undertaken |
| WIN-14 | Wine (2017) | Ferris (2011) ^(78)^ | Not reported in review | Not reported in review | Canada | Not reported in review | Environmental health | Not reported in review | Not undertaken |
| WIN-16 | Wine (2017) | Harding (2012) ^(79)^ | Not reported in review | Not reported in review | USA | Community | Environmental health | Not reported in review | Not undertaken |
| WIN-17 | Wine (2017) | Haynes (2011) ^(80)^ | Not reported in review | Not reported in review | USA | Community | Environmental health | Not reported in review | Not undertaken |
| WIN-18 | Wine (2017) | Israel (1998) ^(81)^ | Not reported in review | Not reported in review | USA | Community | Environmental health | Not reported in review | Not undertaken |
| WIN-19 | Wine (2017) | Israel (2001) | Not reported in review | Not reported in review | USA | Community | Environmental health | Not reported in review | Not undertaken |
| WIN-20 | Wine (2017) | Johnson (2014) ^(82)^ | Not reported in review | Not reported in review | USA | Community | Environmental health | Not reported in review | Not undertaken |
| WIN-21 | Wine (2017) | Matso (2008) ^(83)^ | Not reported in review | Not reported in review | USA | Not reported in review | Environmental health | Not reported in review | Not undertaken |
| WIN-22 | Wine (2017) | McCauley (2001) ^(84)^ | Not reported in review | Not reported in review | USA | Community | Environmental health | Not reported in review | Not undertaken |
| WIN-23 | Wine (2017) | Meadow (2015) ^(85)^ | Not reported in review | Not reported in review | USA | Not reported in review | Environmental health | Not reported in review | Not undertaken |
| WIN-24 | Wine (2017) | Metzler (2003) ^(86)^ | Not reported in review | Not reported in review | USA | Community | Environmental health | Not reported in review | Not undertaken |
| WIN-25 | Wine (2017) | Minkler (2010) ^(87)^ | Not reported in review | Not reported in review | USA | Community | Environmental health | Not reported in review | Not undertaken |
| WIN-26 | Wine (2017) | Nielsen (2001) ^(88)^ | Not reported in review | Not reported in review | Canada | Not reported in review | Environmental health | Not reported in review | Not undertaken |
| WIN-27 | Wine (2017) | Parker (2003) ^(89)^ | Not reported in review | Not reported in review | USA | Community | Environmental health | Not reported in review | Not undertaken |
| WIN-28 | Wine (2017) | Parkes (2004) ^(90)^ | Not reported in review | Not reported in review | New Zealand | Not reported in review | Environmental health | Not reported in review | Not undertaken |
| WIN-29 | Wine (2017) | Pereira (2009) ^(91)^ | Not reported in review | Not reported in review | Italy | Not reported in review | Environmental health | Not reported in review | Not undertaken |
| WIN-30 | Wine (2017) | Ramirez-Andreotta (2014) ^(92)^ | Not reported in review | Not reported in review | USA | Not reported in review | Environmental health | Not reported in review | Not undertaken |
| WIN-31 | Wine (2017) | Ravenscroft (2015) ^(93)^ | Not reported in review | Not reported in review | USA | Community | Environmental health | Not reported in review | Not undertaken |
| WIN-32 | Wine (2017) | Reed (2014) ^(94)^ | Not reported in review | Not reported in review | UK | Not reported in review | Environmental health | Not reported in review | Not undertaken |
| WIN-33 | Wine (2017) | Romero-Lankao (2013) ^(95)^ | Not reported in review | Not reported in review | USA | Not reported in review | Environmental health | Not reported in review | Not undertaken |
| WIN-34 | Wine (2017) | Rosenthal (2007) ^(96)^ | Not reported in review | Not reported in review | USA | Not reported in review | Environmental health | Not reported in review | Not undertaken |
| WIN-35 | Wine (2017) | Schell (1998) ^(97)^ | Not reported in review | Not reported in review | USA | Community | Environmental health | Not reported in review | Not undertaken |
| WIN-36 | Wine (2017) | Schell (2005) ^(98)^ | Not reported in review | Not reported in review | USA | Community | Environmental health | Not reported in review | Not undertaken |
| WIN-37 | Wine (2017) | Schell (2007) ^(99)^ | Not reported in review | Not reported in review | USA | Community | Environmental health | Not reported in review | Not undertaken |
| WIN-38 | Wine (2017) | Strosnider (2014) ^(100)^ | Not reported in review | Not reported in review | USA | Not reported in review | Environmental health | Not reported in review | Not undertaken |
| WIN-39 | Wine (2017) | Wing (2002) ^(101)^ | Not reported in review | Not reported in review | USA | Not reported in review | Environmental health | Not reported in review | Not undertaken |
| WIN-40 | Wine (2017) | Witten (2000) ^(102)^ | Not reported in review | Not reported in review | New Zealand | Not reported in review | Environmental health | Not reported in review | Not undertaken |
| WIN-LOR-01 | Wine (2017), Lorenc (2014) | Gonzalez (2011) ^(103)^ | Process evaluation of community based environmental justice advocacy project | Case studies and interviews | USA | Community | Environmental health; planning | Not reported in review | Score+ (LORENC) |
| WIN-LOR02 | Wine (2017) Lorenc (2014) | Garcia (2013) ^(104)^ | Process evaluation of community-based environmental justice advocacy project | Case studies and interviews | USA | Community | Environmental health | Not reported in review | Score+ (LORENC) |

**Primary study references**

1. Amara N, Ouimet M, Landry R. New Evidence on Instrumental, Conceptual, and Symbolic Utilization of University Research in Government Agencies. Science Communication. 2004; 26:75-106. 10.1177/1075547004267491.

2. Garcia LB, Hernandez KE, Mata H. Professional Development Through Policy Advocacy:Communicating and Advocating for Health and Health Equity. Health Promotion Practice. 2015; 16:162-5. 10.1177/1524839914560405.

3. Giang T, Karpyn A, Laurison HB, Hillier A, Perry RD. Closing the grocery gap in underserved communities: the creation of the Pennsylvania Fresh Food Financing Initiative. J Public Health Manag Pract. 2008; 14:272-9. doi.org/10.1097/01.PHH.0000316486.57512.bf.

4. Gomm M, Lincoln P, Pikora T, Giles-Corti B. Planning and implementing a community-based public health advocacy campaign: a transport case study from Australia. Health Promot Int. 2006; 21:284-92. 10.1093/heapro/dal027.

5. Ingram M, Schachter KA, Sabo SJ, Reinschmidt KM, Gomez S, De Zapien JG, et al. A community health worker intervention to address the social determinants of health through policy change. J Prim Prev. 2014; 35:119-23. doi.org/10.1007/s10935-013-0335-y.

6. McAndrews C, Marcus J. Community-Based Advocacy at the Intersection of Public Health and Transportation:The Challenges of Addressing Local Health Impacts within a Regional Policy Process. Journal of Planning Education and Research. 2014; 34:190-202. doi.org/10.1177/0739456x14531624.

7. Tsoukalas T, Glantz SA. The Duluth clean indoor air ordinance: problems and success in fighting the tobacco industry at the local level in the 21st century. Am J Public Health. 2003; 93:1214-21. 10.2105/ajph.93.8.1214.

8. Brassolotto J, Raphael D, Baldeo N. Epistemological barriers to addressing the social determinants of health among public health professionals in Ontario, Canada: a qualitative inquiry. Critical Public Health. 2014; 24:321-36. doi.org/10.1080/09581596.2013.820256.

9. Roos NP, Roos LL, Brownell M, Fuller EL. Enhancing policymakers' understanding of disparities: relevant data from an information-rich environment. Milbank Q. 2010; 88:382-403. 10.1111/j.1468-0009.2010.00604.x.

10. Izumi BT, Schulz AJ, Israel BA, Reyes AG, Martin J, Lichtenstein RL, et al. The one-pager: a practical policy advocacy tool for translating community-based participatory research into action. Prog Community Health Partnersh. 2010; 4:141-7. doi.org/10.1353/cpr.0.0114.

11. Macnaughton E, Nelson G, Goering P. Bringing politics and evidence together: policy entrepreneurship and the conception of the At Home/Chez Soi Housing First Initiative for addressing homelessness and mental illness in Canada. Soc Sci Med. 2013; 82:100-7. doi.org/10.1016/j.socscimed.2013.01.033.

12. Nelson G. The development of a mental health coalition: a case study. Am J Community Psychol. 1994; 22:229-55. 10.1007/BF02506864.

13. Orton LC, Lloyd-Williams F, Taylor-Robinson DC, Moonan M, O'Flaherty M, Capewell S. Prioritising public health: a qualitative study of decision making to reduce health inequalities. BMC Public Health. 2011; 11:821. 10.1186/1471-2458-11-821.

14. Priest N, Waters E, Valentine N, Armstrong R, Friel S, Prasad A, et al. Engaging policy makers in action on socially determined health inequities: developing evidence-informed cameos. Evidence & Policy: A Journal of Research, Debate and Practice. 2009; 5:53-70. doi.org/10.1332/174426409X395411.

15. Baum FE, Laris P, Fisher M, Newman L, Macdougall C. "Never mind the logic, give me the numbers": former Australian health ministers' perspectives on the social determinants of health. Soc Sci Med. 2013; 87:138-46. doi.org/10.1016/j.socscimed.2013.03.033.

16. Wehrens R, Bekker M, Bal R. The construction of evidence-based local health policy through partnerships: Research infrastructure, process, and context in the Rotterdam 'Healthy in the City' programme. J Public Health Policy. 2010; 31:447-60. 10.1057/jphp.2010.33.

17. Fazli GS, Creatore MI, Matheson FI, Guilcher S, Kaufman-Shriqui V, Manson H, et al. Identifying mechanisms for facilitating knowledge to action strategies targeting the built environment. BMC Public Health. 2017; 17:1. 10.1186/s12889-016-3954-4.

18. King N. Making the case for sport and recreation services. International Journal of Public Sector Management. 2014; 27:152-64. doi.org/10.1108/IJPSM-04-2013-0052.

19. McGill E, Egan M, Petticrew M, Mountford L, Milton S, Whitehead M, et al. Trading quality for relevance: non-health decision-makers' use of evidence on the social determinants of health. BMJ Open. 2015; 5:e007053. doi.org/10.1136/bmjopen-2014-007053.

20. Milton S, Petticrew M, Green J. Why do local authorities undertake controlled evaluations of health impact? A qualitative case study of interventions in housing. Public Health. 2014; 128:1112-7. doi.org/10.1016/j.puhe.2014.10.009.

21. Phillips G, Green J. Working for the public health: politics, localism and epistemologies of practice. Sociol Health Illn. 2015; 37:491-505. doi.org/10.1111/1467-9566.12214.

22. Willmott M, Womack J, Hollingworth W, Campbell R. Making the case for investment in public health: experiences of Directors of Public Health in English local government. J Public Health (Oxf). 2016; 38:237-42. 10.1093/pubmed/fdv035.

23. Blackman T, Harrington B, Elliott E, Greene A, Hunter DJ, Marks L, et al. Framing health inequalities for local intervention: comparative case studies. Sociol Health Illn. 2012; 34:49-63. doi.org/10.1111/j.1467-9566.2011.01362.x.

24. Allender S, Cavill N, Parker M, Foster C. ‘Tell us something we don't already know or do!’ — The response of planning and transport professionals to public health guidance on the built environment and physical activity. Journal of Public Health Policy. 2009; 30:102-16. 10.1057/jphp.2008.43.

25. Allison KW, Edmonds T, Wilson K, Pope M, Farrell AD. Connecting youth violence prevention, positive youth development, and community mobilization. Am J Community Psychol. 2011; 48:8-20. doi.org/10.1007/s10464-010-9407-9.

26. Coote A, Allen J, Woodhead D. Finding Out What Works. Building Knowledge About Complex, Community-Based Initiatives. London, UK: The Kings Fund2004.

27. Freeman E, Thompson S, Jalaludin B. Healthy built environments: stakeholder engagement in evidence based policy making. In: Whitzman C, Fincher R, editors. Proceedings of the State of Australian Cities Conference; 29 November–2 December; Melbourne, Australia: Australian Sustainable Cities and Regions Network; 2011.

28. Gudmundsson H, Ericsson E, Tight M, Lawler M, Envall P, Figueroa MJ, et al. The Role of Decision Support in the Implementation of “Sustainable Transport” Plans. European Planning Studies. 2012; 20:171-91. 10.1080/09654313.2012.650903.

29. Harrison C, Davies G. Conserving biodiversity that matters: practitioners' perspectives on brownfield development and urban nature conservation in London. Journal of Environmental Management. 2002; 65:95-108. <https://doi.org/10.1006/jema.2002.0539>.

30. Hatzopoulou M, Miller EJ. Transport policy evaluation in metropolitan areas: The role of modelling in decision-making. Transportation Research Part A: Policy and Practice. 2009; 43:323-38. <https://doi.org/10.1016/j.tra.2008.11.001>.

31. Hewson P. Evidence-based practice in road casualty reduction. Inj Prev. 2007; 13:291-2. 10.1136/ip.2007.015214.

32. Hinchcliff R, Poulos R, Ivers RQ, Senserrick T. Understanding novice driver policy agenda setting. Public Health. 2011; 125:217-21. 10.1016/j.puhe.2011.01.001.

33. Marsden G, Frick KT, May AD, Deakin E. How do cities approach policy innovation and policy learning? A study of 30 policies in Northern Europe and North America. Transport Policy. 2011; 18:501-12. <https://doi.org/10.1016/j.tranpol.2010.10.006>.

34. Minkler M, Garcia AP, Williams J, LoPresti T, Lilly J. Sí se puede: using participatory research to promote environmental justice in a Latino community in San Diego, California. J Urban Health. 2010; 87:796-812. 10.1007/s11524-010-9490-0.

35. Petersen D, Minkler M, Vásquez VB, Baden AC. Community-Based Participatory Research as a Tool for Policy Change: A Case Study of the Southern California Environmental Justice Collaborative. Review of Policy Research. 2006; 23:339-54. <https://doi.org/10.1111/j.1541-1338.2006.00204.x>.

36. Sandström UG, Angelstam P, Khakee A. Urban comprehensive planning – identifying barriers for the maintenance of functional habitat networks. Landscape and Urban Planning. 2006; 75:43-57. <https://doi.org/10.1016/j.landurbplan.2004.11.016>.

37. Timms P. Urban transport policy transfer: “bottom-up” and “top-down” perspectives. Transport Policy. 2011; 18:513-21. <https://doi.org/10.1016/j.tranpol.2010.10.009>.

38. Armstrong R, Waters E, Moore L, Dobbins M, Pettman T, Burns C, et al. Understanding evidence: a statewide survey to explore evidence-informed public health decision-making in a local government setting. Implement Sci. 2014; 9:188. doi.org/10.1186/s13012-014-0188-7.

39. Francis D, Turley R, Thomson H, Weightman A, Waters E, Moore L. Supporting the needs of public health decision-makers and review authors in the UK. J Public Health (Oxf). 2015; 37:172-4. doi.org/10.1093/pubmed/fdu089.

40. Jardine C, Furgal C, Garvin T, McGee T. Factors affecting the communication and understanding of health risks innorthern aboriginal communities in Canada. Int J Psychol. 2004; 39:518.

41. Pufall EL, Jones AQ, McEwen SA, Lyall C, Peregrine AS, Edge VL. Community-derived research dissemination strategies in an Inuit community. Int J Circumpolar Health. 2011; 70:532-41. doi.org/10.3402/ijch.v70i5.17860.

42. Tyrrell M. Making Sense of Contaminants: A Case Study of Arviat, Nunavut. Arctic. 2006; 59:370-80.

43. Armstrong R, Doyle J, Lamb C, Waters E. Multi-sectoral health promotion and public health: the role of evidence. Journal of Public Health. 2006; 28:168-72. doi.org/10.1093/pubmed/fdl013.

44. Jennings ET, Jr., Hall JL. Evidence-Based Practice and the Use of Information in State Agency Decision Making. Journal of Public Administration Research and Theory. 2011; 22:245-66. 10.1093/jopart/mur040.

45. Lavis JN, Farrant MS, Stoddart GL. Barriers to employment-related healthy public policy in Canada. Health Promot Int. 2001; 16:9-20. 10.1093/heapro/16.1.9.

46. Smith KE, Joyce KE. Capturing complex realities: understanding efforts to achieve evidence-based policy and practice in public health. Evidence & Policy: A Journal of Research, Debate and Practice. 2012; 8:57-78. 10.1332/174426412X6201371.

47. Weitkamp G, Van den Berg AE, Bregt AK, Van Lammeren RJ. Evaluation by policy makers of a procedure to describe perceived landscape openness. J Environ Manage. 2012; 95:17-28. 10.1016/j.jenvman.2011.09.022.

48. Bickford JJ, Kothari AR. Research and knowledge in Ontario tobacco control networks. Can J Public Health. 2008; 99:297-300.

49. Jack SM, Brooks S, Furgal CM, Dobbins M. Knowledge transfer and exchange processes for environmental health issues in Canadian Aboriginal communities. Int J Environ Res Public Health. 2010; 7:651-74. doi.org/10.3390/ijerph7020651.

50. Green J. Epistemology, evidence and experience: evidence based health care in the work of Accident Alliances. Sociology of Health & Illness. 2000; 22:453-76. <https://doi.org/10.1111/1467-9566.00214>.

51. Macintyre S, Chalmers I, Horton R, Smith R. Using evidence to inform health policy: case study. BMJ. 2001; 322:222-5. doi.org/10.1136/bmj.322.7280.222.

52. Taylor-Robinson DC, Milton B, Lloyd-Williams F, O'Flaherty M, Capewell S. Planning ahead in public health? A qualitative study of the time horizons used in public health decision-making. BMC Public Health. 2008; 8:415. 10.1186/1471-2458-8-415.

53. Baum F, Newman L, Biedrzycki K, Patterson J. Can a regional government's social inclusion initiative contribute to the quest for health equity? Health Promot Int. 2010; 25:474-82. doi.org/10.1093/heapro/daq033.

54. Cacari-Stone L, Wallerstein N, Garcia AP, Minkler M. The promise of community-based participatory research for health equity: a conceptual model for bridging evidence with policy. Am J Public Health. 2014; 104:1615-23. doi.org/10.2105/AJPH.2014.301961.

55. Knight EK. Shifting public health practice to advance health equity: recommendations from experts and community leaders. J Public Health Manag Pract. 2014; 20:188-96. doi.org/10.1097/PHH.0b013e31829959fb.

56. Raphael D, Brassolotto J, Baldeo N. Ideological and organizational components of differing public health strategies for addressing the social determinants of health. Health Promot Int. 2015; 30:855-67. doi.org/10.1093/heapro/dau022.

57. Raphael D, Brassolotto J. Understanding action on the social determinants of health: a critical realist analysis of in-depth interviews with staff of nine Ontario public health units. BMC Res Notes. 2015; 8:105. 10.1186/s13104-015-1064-5.

58. Israel BA, Schulz AJ, Estrada-Martinez L, Zenk SN, Viruell-Fuentes E, Villarruel AM, et al. Engaging urban residents in assessing neighborhood environments and their implications for health. J Urban Health. 2006; 83:523-39. doi.org/10.1007/s11524-006-9053-6.

59. Israel BA, Lichtenstein R, Lantz P, McGranaghan R, Allen A, Guzman JR, et al. The Detroit Community-Academic Urban Research Center: development, implementation, and evaluation. J Public Health Manag Pract. 2001; 7:1-19. doi.org/10.1097/00124784-200107050-00003.

60. Schulz AJ, Israel BA, Parker EA, Lockett M, Hill Y, Wills R. The East Side Village Health Worker Partnership: integrating research with action to reduce health disparities. Public Health Rep. 2001; 116:548-57. 10.1093/phr/116.6.548.

61. Schulz AJ, Parker EA, Israel BA, Allen A, Decarlo M, Lockett M. Addressing social determinants of health through community-based participatory research: the East Side Village Health Worker Partnership. Health Educ Behav. 2002; 29:326-41. 10.1177/109019810202900305.

62. Israel BA, Parker EA, Rowe Z, Salvatore A, Minkler M, Lopez J, et al. Community-based participatory research: lessons learned from the Centers for Children's Environmental Health and Disease Prevention Research. Environ Health Perspect. 2005; 113:1463-71. doi.org/10.1289/ehp.7675.

63. Minkler M, Vásquez VB, Shepard P. Promoting Environmental Health Policy Through Community Based Participatory Research: A Case Study from Harlem, New York. Journal of Urban Health. 2006; 83:101-10. doi.org/10.1007/s11524-005-9010-9.

64. Minkler M, Vásquez VB, Tajik M, Petersen D. Promoting environmental justice through community-based participatory research: the role of community and partnership capacity. Health Educ Behav. 2008; 35:119-37. doi.org/10.1177/1090198106287692.

65. Angelstam P, Andersson K, Annerstedt M, Axelsson R, Elbakidze M, Garrido P, et al. Solving problems in social-ecological systems: definition, practice and barriers of transdisciplinary research. Ambio. 2013; 42:254-65. 10.1007/s13280-012-0372-4.

66. Arcury TA, Quandt SA, Dearry A. Farmworker pesticide exposure and community-based participatory research: rationale and practical applications. Environ Health Perspect. 2001; 109 429-34. doi.org/10.1289/ehp.01109s3429.

67. Austin D. Confronting Environmental Challenges on the US–Mexico Border: Long-Term Community-Based Research and Community Service Learning in a Binational Partnership. Journal of Community Practice. 2010; 18:361-95. 10.1080/10705422.2010.490112.

68. Bharadwaj L. A framework for building research partnerships with first nations communities. Environ Health Insights. 2014; 8:15-25. 10.4137/EHI.S10869.

69. Boon WPC, Chappin MMH, Perenboom J. Balancing divergence and convergence in transdisciplinary research teams. Environmental Science & Policy. 2014; 40:57-68. <https://doi.org/10.1016/j.envsci.2014.04.005>.

70. Brown P, Brody JG, Morello-Frosch R, Tovar J, Zota AR, Rudel RA. Measuring the success of community science: the northern California Household Exposure Study. Environ Health Perspect. 2012; 120:326-31. 10.1289/ehp.1103734.

71. Burger J, Gochfeld M, Powers CW, Kosson DS, Halverson J, Siekaniec G, et al. Scientific research, stakeholders, and policy: continuing dialogue during research on radionuclides on Amchitka Island, Alaska. J Environ Manage. 2007; 85:232-44. 10.1016/j.jenvman.2006.10.005.

72. Burger J, Gochfeld M, Pletnikoff K. Collaboration versus communication: The Department of Energy's Amchitka Island and the Aleut Community. Environ Res. 2009; 109:503-10. 10.1016/j.envres.2009.01.002.

73. Collman GW. Community-based approaches to environmental health research around the globe. Rev Environ Health. 2014; 29:125-8. 10.1515/reveh-2014-0030.

74. Conrad PA, Meek LA, Dumit J. Operationalizing a One Health approach to global health challenges. Comp Immunol Microbiol Infect Dis. 2013; 36:211-6. 10.1016/j.cimid.2013.03.006.

75. Corburn J. Community knowledge in environmental health science: co-producing policy expertise. Environmental Science & Policy. 2007; 10:150-61. <https://doi.org/10.1016/j.envsci.2006.09.004>.

76. Cummins C, Doyle J, Kindness L, Lefthand MJ, Bear Dont Walk UJ, Bends AL, et al. Community-based participatory research in Indian country: improving health through water quality research and awareness. Fam Community Health. 2010; 33:166-74. 10.1097/FCH.0b013e3181e4bcd8.

77. Downs TJ, Ross L, Patton S, Rulnick S, Sinha D, Mucciarone D, et al. Complexities of holistic community-based participatory research for a low income, multi-ethnic population exposed to multiple built-environment stressors in Worcester, Massachusetts. Environ Res. 2009; 109:1028-40. 10.1016/j.envres.2009.08.005.

78. Ferris LE, Sass-Kortsak A. Sharing research findings with research participants and communities. Int J Occup Environ Med. 2011; 2:172-81.

79. Harding A, Harper B, Stone D, O'Neill C, Berger P, Harris S, et al. Conducting research with tribal communities: sovereignty, ethics, and data-sharing issues. Environ Health Perspect. 2012; 120:6-10. 10.1289/ehp.1103904.

80. Haynes EN, Beidler C, Wittberg R, Meloncon L, Parin M, Kopras EJ, et al. Developing a bidirectional academic-community partnership with an Appalachian-American community for environmental health research and risk communication. Environ Health Perspect. 2011; 119:1364-72. 10.1289/ehp.1003164.

81. Israel BA, Schulz AJ, Parker EA, Becker AB. Review of community-based research: assessing partnership approaches to improve public health. Annu Rev Public Health. 1998; 19:173-202. 10.1146/annurev.publhealth.19.1.173.

82. Johnson S, Cardona D, Gramling B, Hamilton C, Hoffmann R, Sabir J. Engaging the for-profit sector in community-based participatory research: lessons from the ground. Prog Community Health Partnersh. 2014; 8:523-30. 10.1353/cpr.2014.0062.

83. Matso KE, Dix MO, Chicoski B, Hernandez DL, Schubel JR. Establishing a minimum standard for collaborative research in federal environmental agencies. Integr Environ Assess Manag. 2008; 4:362-8. 10.1897/IEAM_2007-070.1.

84. McCauley LA, Beltran M, Phillips J, Lasarev M, Sticker D. The Oregon migrant farmworker community: an evolving model for participatory research. Environ Health Perspect. 2001; 109 Suppl 3:449-55. 10.1289/ehp.01109s3449.

85. Meadow AM, Ferguson DB, Guido Z, Horangic A, Owen G, Wall T. Moving toward the Deliberate Coproduction of Climate Science Knowledge. Weather, Climate, and Society. 2015; 7:179-91. 10.1175/wcas-d-14-00050.1.

86. Metzler MM, Higgins DL, Beeker CG, Freudenberg N, Lantz PM, Senturia KD, et al. Addressing urban health in Detroit, New York City, and Seattle through community-based participatory research partnerships. Am J Public Health. 2003; 93:803-11. 10.2105/ajph.93.5.803.

87. Minkler M. Linking Science and Policy Through Community-Based Participatory Research to Study and Address Health Disparities. American Journal of Public Health. 2010; 100:S81-S7. 10.2105/ajph.2009.165720.

88. Nielsen NO. Ecosystem approaches to human health. Cad Saude Publica. 2001; 17 Suppl:69-75. 10.1590/s0102-311x2001000700015.

89. Parker EA, Israel BA, Williams M, Brakefield-Caldwell W, Lewis TC, Robins T, et al. Community action against asthma: examining the partnership process of a community-based participatory research project. J Gen Intern Med. 2003; 18:558-67. 10.1046/j.1525-1497.2003.20322.x.

90. Parkes M, Eyles R, Benwell G. Integration of ecology and health research at the catchment scale: The Taieri River catchment, New Zealand. Journal of Rural and Remote Environmental Health. 2004; 3:1-17.

91. Guimarães Pereira Â, Raes F, De Sousa Pedrosa T, Rosa P, Brodersen S, Jørgensen MS, et al. Atmospheric composition change research: Time to go post-normal? Atmospheric Environment. 2009; 43:5423-32. <https://doi.org/10.1016/j.atmosenv.2009.06.016>.

92. Ramirez-Andreotta MD, Brusseau ML, Artiola JF, Maier RM, Gandolfi AJ. Environmental Research Translation: enhancing interactions with communities at contaminated sites. Sci Total Environ. 2014; 497-498:651-64. 10.1016/j.scitotenv.2014.08.021.

93. Ravenscroft J, Schell LM, Cole T. Applying the community partnership approach to human biology research. Am J Hum Biol. 2015; 27:6-15. 10.1002/ajhb.22652.

94. Reed MS, Stringer LC, Fazey I, Evely AC, Kruijsen JHJ. Five principles for the practice of knowledge exchange in environmental management. J Environ Manage. 2014; 146:337-45. 10.1016/j.jenvman.2014.07.021.

95. Romero-Lankao P, Borbor-Cordova M, Abrutsky R, Günther G, Behrentz E, Dawidowsky L. ADAPTE: A tale of diverse teams coming together to do issue-driven interdisciplinary research. Environmental Science & Policy. 2013; 26:29-39. <https://doi.org/10.1016/j.envsci.2011.12.003>.

96. Rosenthal JK, Sclar ED, Kinney PL, Knowlton K, Crauderueff R, Brandt-Rauf PW. Links between the built environment, climate and population health: interdisciplinary environmental change research in New York City. Ann Acad Med Singap. 2007; 36:834-46.

97. Schell LM, Tarbell AM. A partnership study of PCBs and the health of Mohawk youth: lessons from our past and guidelines for our future. Environ Health Perspect. 1998; 106 Suppl 3:833-40. 10.1289/ehp.98106833.

98. Schell LM, Ravenscroft J, Cole M, Jacobs A, Newman J, Akwesasne Task Force On The E. Health disparities and toxicant exposure of Akwesasne Mohawk young adults: a partnership approach to research. Environ Health Perspect. 2005; 113:1826-32. 10.1289/ehp.7914.

99. Schell LM, Ravenscroft J, Gallo M, Denham M. Advancing biocultural models by working with communities: a partnership approach. Am J Hum Biol. 2007; 19:511-24. 10.1002/ajhb.20611.

100. Strosnider H, Zhou Y, Balluz L, Qualters J. Engaging academia to advance the science and practice of environmental public health tracking. Environ Res. 2014; 134:474-81. 10.1016/j.envres.2014.04.039.

101. Wing S. Social responsibility and research ethics in community-driven studies of industrialized hog production. Environ Health Perspect. 2002; 110:437-44. 10.1289/ehp.02110437.

102. Witten K, Parkes M, Ramasubramanian L. Participatory environmental health research in Aotearoa/New Zealand: constraints and opportunities. Health Educ Behav. 2000; 27:371-84. 10.1177/109019810002700311.

103. Gonzalez PA, Minkler M, Garcia AP, Gordon M, Garzon C, Palaniappan M, et al. Community-based participatory research and policy advocacy to reduce diesel exposure in West Oakland, California. Am J Public Health. 2011; 101 Suppl 1:S166-75. 10.2105/AJPH.2010.196204.

104. Garcia AP, Wallerstein N, Hricko A, Marquez JN, Logan A, Green Nasser E, et al. THE (Trade, Health, Environment) Impact Project: A Community-Based Participatory Research Environmental Justice Case Study. Environmental Justice. 2013; 6:17-26. 10.1089/env.2012.0016.
